# Prediabetes and risk of mortality, diabetes-related complications and comorbidities: umbrella review of meta-analyses of prospective studies

**DOI:** 10.1007/s00125-021-05592-3

**Published:** 2021-10-31

**Authors:** Sabrina Schlesinger, Manuela Neuenschwander, Janett Barbaresko, Alexander Lang, Haifa Maalmi, Wolfgang Rathmann, Michael Roden, Christian Herder

**Affiliations:** 1 Institute for Biometrics and Epidemiology, German Diabetes Center (Deutsches Diabetes-Zentrum/DDZ), Leibniz Center for Diabetes Research at Heinrich Heine University Düsseldorf, Düsseldorf, Germany; 2 German Center for Diabetes Research (DZD), Partner Düsseldorf, Düsseldorf, Germany; 3 Institute for Clinical Diabetology, German Diabetes Center (Deutsches Diabetes-Zentrum/DDZ), Leibniz Center for Diabetes Research at Heinrich Heine University Düsseldorf, Düsseldorf, Germany; 4 Department of Endocrinology and Diabetology, Medical Faculty and University Hospital, Heinrich Heine University, Düsseldorf, Germany

**Keywords:** Complications, Meta-analysis, Mortality, Prediabetes, Systematic review, Umbrella review

## Abstract

**Aims/hypothesis:**

The term prediabetes is used for individuals who have impaired glucose metabolism whose glucose or HbA_1c_ levels are not yet high enough to be diagnosed as diabetes. Prediabetes may already be associated with an increased risk of chronic ‘diabetes-related’ complications. This umbrella review aimed to provide a systematic overview of the available evidence from meta-analyses of prospective observational studies on the associations between prediabetes and incident diabetes-related complications in adults and to evaluate their strength and certainty.

**Methods:**

For this umbrella review, systematic reviews with meta-analyses reporting summary risk estimates for the associations between prediabetes (based on fasting or 2 h postload glucose or on HbA_1c_) and incidence of diabetes-related complications, comorbidities and mortality risk were included. PubMed, Web of Science, the Cochrane Library and Epistemonikos were searched up to 17 June 2021. Summary risk estimates were recalculated using a random effects model. The certainty of evidence was evaluated by applying the GRADE tool. This study is registered with PROSPERO, CRD42020153227.

**Results:**

Ninety-five meta-analyses from 16 publications were identified. In the general population, prediabetes was associated with a 6–101% increased risk for all-cause mortality and the incidence of cardiovascular outcomes, CHD, stroke, heart failure, atrial fibrillation and chronic kidney disease, as well as total cancer, total liver cancer, hepatocellular carcinoma, breast cancer and all-cause dementia with moderate certainty of evidence. No associations between prediabetes and incident depressive symptoms and cognitive impairment were observed (with low or very low certainty of evidence). The association with all-cause mortality was stronger for prediabetes defined by impaired glucose tolerance than for prediabetes defined by HbA_1c_.

**Conclusions/interpretation:**

Prediabetes was positively associated with risk of all-cause mortality and the incidence of cardiovascular outcomes, CHD, stroke, chronic kidney disease, cancer and dementia. Further high-quality studies, particularly on HbA_1c_-defined prediabetes and other relevant health outcomes (e. g. neuropathy) are required to support the evidence.

**Graphical abstract:**

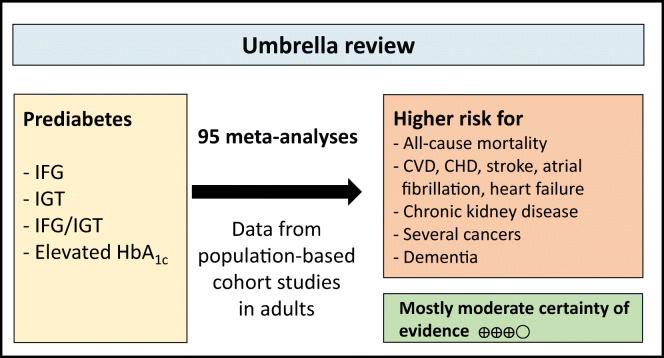

**Supplementary Information:**

The online version contains peer-reviewed but unedited supplementary material available at 10.1007/s00125-021-05592-3.

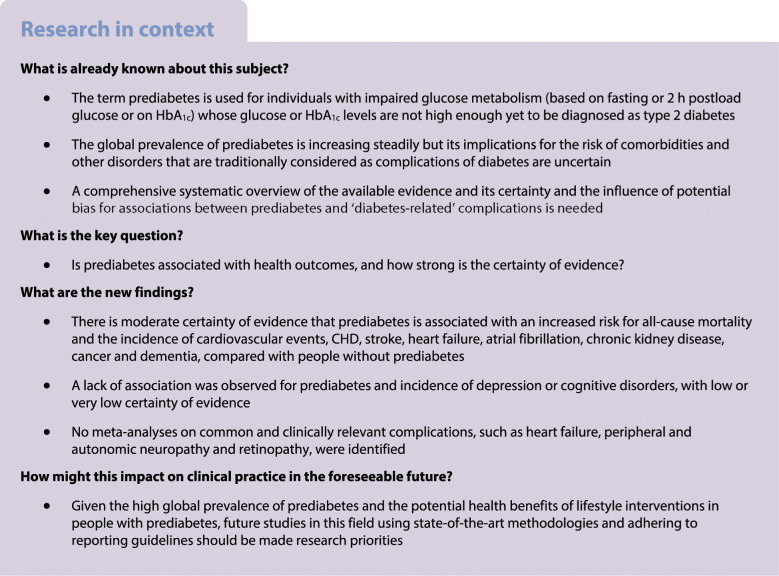



## Introduction

The term ‘prediabetes’ is used for individuals with impaired glucose metabolism not fulfilling the diagnostic criteria of type 2 diabetes. Based on the recent guidelines from the ADA, this definition is met if at least one of the following criteria applies: impaired fasting glucose (IFG) (fasting plasma glucose 5.6–6.9 mmol/l); impaired glucose tolerance (IGT) (2 h glucose during a 75 g OGTT 7.8–11.0 mmol/l); or HbA_1c_ in the range of 39–47 mmol/mol (5.7–6.4%) [1]. The IDF estimated the prevalence of IGT in the adult population to be 7.5% (373.9 million) in 2019 and projected an increase to 8.6% (548.4 million) in 2045 [2]. A recent study in China used fasting plasma glucose and HbA_1c_ to define prediabetes and found a prevalence of 35.7% among the Chinese adult population [3].

The concept of prediabetes is important because of its high prevalence and the high risk of progression to overt type 2 diabetes among individuals with prediabetes [1, 4]. However, various metabolic abnormalities already exist before the onset of diabetes [5] and may confer an increased risk of multiple comorbidities and chronic complications that are traditionally seen as ‘diabetes-related’ [2, 6]. Meta-analyses have linked prediabetes with higher risk of cardiovascular outcomes and chronic kidney disease [7, 8]. In addition, there is emerging evidence that prediabetes is associated with the development of less frequently assessed comorbidities of diabetes such as cognitive disorders and cancer [9, 10]. A recent study from the UK estimated that half of the study population already had a macro- or microvascular disease at the time of type 2 diabetes diagnosis [11].

Given these findings, prediabetes represents a time window of opportunity in which modifiable risk factors, such as overweight/obese state, diet high in energy and physical inactivity, can be targeted to prevent or delay the development of type 2 diabetes [12, 13]. Lifestyle intervention in people with prediabetes may also have beneficial effects on the development of CVD, microvascular complications and both cardiovascular and all-cause mortality in the long-term [14]. However, this is controversial given the lack of studies with appropriate sample sizes and follow-up times [15].

To estimate the full clinical relevance of prediabetes as a potential indication for intervention measures, clarification is needed regarding the health outcomes for which prediabetes is a risk factor and the strength of evidence for these associations. In addition, potential biases of the published reports and research gaps in the literature need to be evaluated. In this context, umbrella reviews are helpful tools because they provide a broad overview of the existing evidence by focusing on published systematic reviews with meta-analyses on a specific topic and enable evaluation of the certainty of evidence and the risk of bias of the published meta-analyses [16].

Therefore, this study aimed to conduct an umbrella review of systematic reviews with meta-analyses to provide a comprehensive overview of the available evidence from prospective studies on the association between prediabetes and the incidence of diabetes-related complications and comorbidities in adults and to evaluate the strength and certainty of this evidence.

## Methods

### Data sources and searches

This umbrella review was conducted and reported according to the PRISMA statement and to ‘Preferred reporting items for overviews of systematic reviews’ [17, 18]. A protocol was prospectively registered at PROSPERO (CRD42020153227).

The systematic literature search was conducted from inception up to 17 June 2021 in PubMed, Web of Science, the Cochrane Library and Epistemonikos by using a pre-defined search term (electronic supplementary material [ESM] Table 1). We did not apply any restrictions or filters. To identify further relevant articles, we screened the reference lists of the identified reports. The literature screening was conducted independently by at least two authors. Disagreements were resolved by consensus.

### Study selection

Systematic reviews with meta-analyses from prospective studies on prediabetes and incidence of diabetes-related complications, comorbidities or risk of mortality were included if they met the following eligibility criteria: prediabetes had to be defined according to blood glucose or HbA_1c_ levels that were higher than normal but not high enough to be diagnosed as diabetes. We accepted the diagnostic criteria from the ADA, WHO and IDF (Table 1). The comparators were defined as no prediabetes according to blood glucose levels or HbA_1c_ in the normal range. We only considered meta-analyses that were based on the following inclusion criteria: adult participants (≥18 years); study sample from the general population or patient groups (e.g. with CVD); and absence of diagnosed diabetes or gestational diabetes at baseline. Meta-analyses on children, adolescents or pregnant women were excluded (we are not aware of any meta-analyses or identified meta-analyses during our search that focused on these groups). Regarding the outcomes, we considered incidence of any diabetes-related complications, comorbidities or mortality risk without restrictions. Systematic reviews without a meta-analysis were excluded. If a report presented more than one meta-analysis (multiple outcomes [e.g. risk of all-cause mortality, CVD mortality, stroke, etc.]), all meta-analyses were included separately. If reports focused on the same outcome but used different prediabetes definitions, all reports were included as single meta-analyses. If multiple reports were available for the meta-analysis (same definition of prediabetes and same outcome), we selected the largest meta-analysis with the largest number of primary studies and/or the report that provided the most detailed information (e.g. meta-analyses separately for the general population and participant groups).

**Table 1:** Definitions of prediabetes

Definition	ADA	WHO	IDF
	Prediabetes	Intermediate hyperglycaemia	IFG, IGT
IFG			
FPG in mmol/l	5.6–6.9	6.1–6.9	6.1–6.9
FPG in mg/dl	100–125	110–125	110–125
IGT			
2 h PG in mmol/l	7.8–11.0	7.8–11.0	7.8–11.0
2 h PG in mg/dl	140–199	140–199	140–199
HbA_1c_			–
mmol/mol	39–47		–
%	5.7–6.4		–

2 h PG, 2 h plasma glucose during 75 g OGTT; FPG, fasting plasma glucose

### Data extraction and quality assessment

One author extracted relevant data and another investigator double-checked extracted data for accuracy. Any discrepancies were discussed and resolved by discussion. For each published meta-analysis, the data listed in ESM Table 2 were extracted.

The methodological quality was evaluated by applying the ROBIS tool [19]. This tool consists of four domains: (1) study eligibility criteria; (2) identification and selection of studies; (3) data collection and study appraisal; and (4) synthesis and findings. Finally, an overall evaluation (high or low risk of bias) considering the identified bias along with the interpretation of findings and discussion of the limitations was done. At least two researchers independently assessed the risk of bias of each meta-analysis, and discrepancies were resolved by discussion.

### Data synthesis and analysis

The summary HRs (SHRs) and their corresponding 95% CIs for the association of prediabetes (vs blood glucose levels or HbA_1c_ in the normal range) with diabetes-related complications were recalculated using the random effects model of DerSimonian and Laird [20]. When an original meta-analysis presented results from the same cohort separately (e.g. by sex), we combined the summary effects per cohort first, using a fixed effect model, before conducting the overall meta-analysis. If a published meta-analysis included a study with a prediabetes definition that was not in accordance with our definition, we excluded this primary study in our recalculations. In addition, if a published meta-analysis combined primary studies with different prediabetes definitions, we additionally recalculated this meta-analysis for each prediabetes definition separately whenever sufficient data were available. Heterogeneity was evaluated by calculating *I*^2^, τ^2^ and 95% prediction intervals. Small study effects and publication bias were assessed by using graphical (funnel plots) and statistical tests (Egger’s test), if ≥10 studies were available [21]. A *p* value of <0.10 indicated the presence of publication bias. All statistical analyses were performed using Stata (version 15; Stata-Corp, College Station, TX, USA).

### Certainty of evidence

The certainty of evidence was evaluated by two authors independently using the GRADE tool [22]. Observational studies start with a low certainty of evidence because of risk of bias due to residual confounding. Indications of inconsistency across studies (differences of point estimates, non-overlap of 95% CIs and *I*^2^ statistics), indirectness (e.g. mixed outcomes), imprecision of the findings (precision of the 95% CI and the inclusion of appreciable benefit and harm) and the presence of publication bias can lead to downgrading. On the other hand, a large effect (SHR either ≥2.0 or ≤0.5) and a dose–response gradient can lead to upgrading. The specific reasons for downgrading are provided with the results. The overall rating categorises the certainty of evidence into high, moderate, low or very low. High and moderate certainty in evidence means that it is very likely or probable that the true effect lies close to the estimated finding, and low or very low means that we have little or very little confidence in the finding.

## Results

**Fig. 1:**
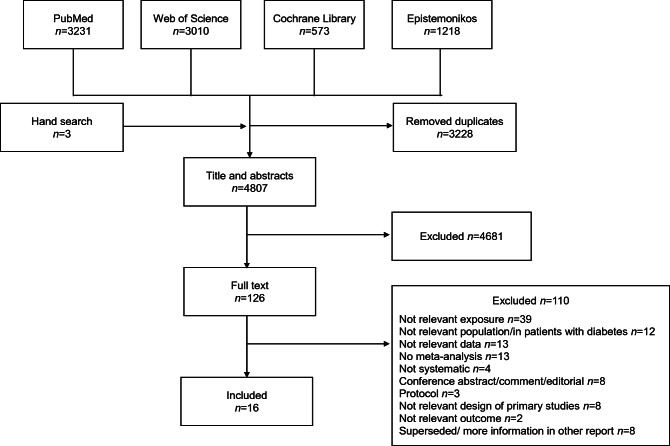
Flow chart of the study selection process

**Fig. 2:**
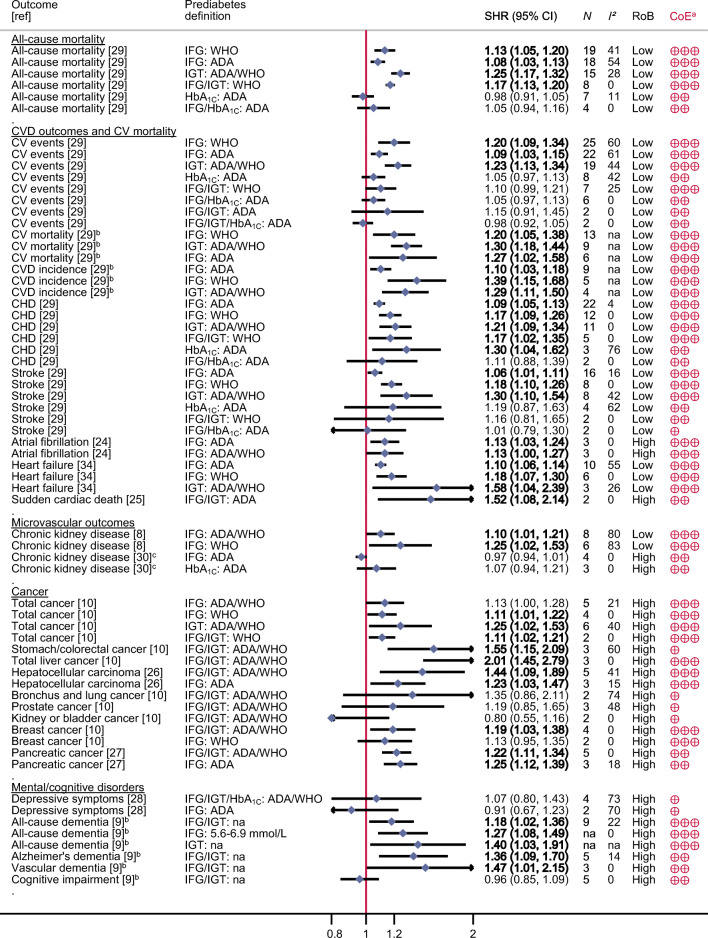
Associations between prediabetes and risk of diabetes-related complications, comorbidities and mortality among the general population. ^a^Interpretation of the certainty of evidence is denoted by crossed circles: four symbols, high; three symbols, moderate; two symbols, low; and one symbol, very low. ^b^Could not be recalculated because of missing information. ^c^Individuals with diabetes at follow-up were excluded. Boldface indicates that the 95% CI does not include the null value and findings are precisely estimated. CoE, certainty of evidence; CV, cardiovascular; *N*, number of primary studies; na, not available; RoB, risk of bias

**Fig. 3:**
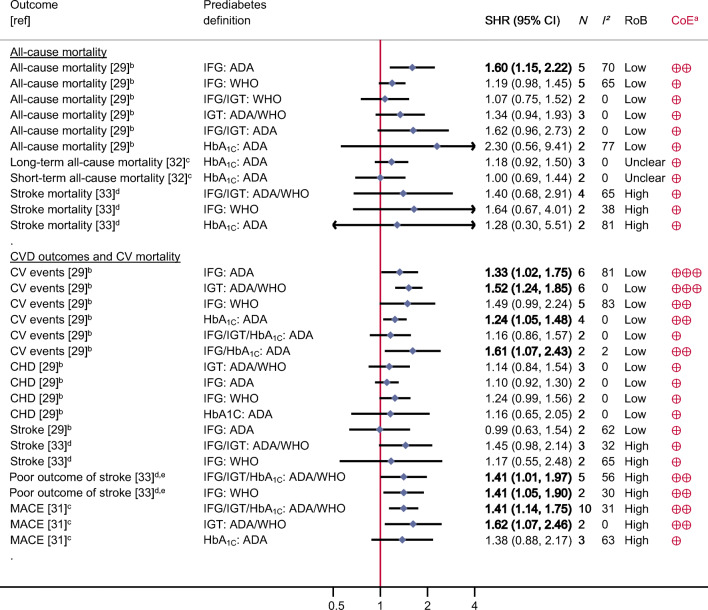
Associations between prediabetes and risk of CVD, cardiovascular mortality and overall mortality among patients with pre-existing diseases. ^a^Interpretation of the certainty of evidence is denoted by crossed circles: four symbols, high; three symbols, moderate; two symbols, low; and one symbol, very low. ^b^Patients with atherosclerotic CVD. ^c^Patients after percutaneous coronary intervention. ^d^Patients with history of stroke/transient ischaemic attack. ^e^Poor outcome of stroke defined as degree of disability or dependence in the daily activities of people who have suffered a stroke or other causes of neurological disability. Boldface indicates that the 95% CI does not include the null value and findings are precisely estimated. CoE, certainty of evidence; CV, cardiovascular; *N*, number of primary studies; MACE, major adverse cardiac events; RoB: Risk of bias

Out of the 4807 identified articles, 126 full-texts were assessed for eligibility, and finally, 16 articles were included in this umbrella review (Fig. 1) [7–10, 23–34]. A detailed list of the excluded articles and the reasons for their exclusion is displayed in ESM Table 3. Out of the included articles, 13 focused on individuals from the general population [7–10, 23–30, 34] and four on specific patient groups, including those with CVD [29], patients after percutaneous coronary intervention [31, 32] or in individuals with a history of stroke/transient ischaemic attack [33]. The 16 articles comprised 95 meta-analyses. Figures 2 and 3 and ESM Table 4 list all outcomes for which associations with prediabetes were identified.

The number of the included primary studies in the meta-analyses ranged from two to 25, and these studies were conducted in the USA, Europe, Asia and Oceania. Prediabetes was defined by using different classifications and cut-off values for IFG, IGT and HbA_1c_ defined by the ADA or WHO. A detailed description of the meta-analyses is shown in ESM Table 4. Most of the primary studies adjusted for several important confounders (age, sex, BMI/waist circumference, smoking, BP/treatment of hypertension and blood lipids/treatment of hyperlipidaemia) (ESM Table 5).

The methodological quality was rated as low risk of bias for four reports [7, 8, 29, 34] and high risk of bias for the remaining reports. Major sources of bias were as follows: an unclear definition of prediabetes; the use of filters in the search (e.g. restricted to human studies); lack of clarity regarding whether screening of the studies, data extraction and/or assessment of the risk of bias of the studies were conducted independently by at least two investigators; and complete lack of the assessment of risk of bias and/or its discussion. A detailed description is shown in ESM Table 6.

Figure 2 summarises the SHRs with 95% CIs of each identified meta-analysis of data for the general population. Whenever possible, the meta-analyses are shown separately by the different definitions of prediabetes (IFG, IGT or HbA_1c_ with cut-offs provided by the ADA or WHO). There was moderate certainty of evidence for an association between prediabetes (IFG defined by the ADA and WHO and/or IGT) and increased risk of all-cause mortality, cardiovascular events (incidence and mortality combined), CVD mortality, CVD incidence, CHD, stroke, heart failure, atrial fibrillation, chronic kidney disease, total cancer, total liver cancer (liver and intrahepatic bile ducts), hepatocellular carcinoma, breast cancer and all-cause dementia (ESM Table 7). The relative risk of the associations ranged from 6 to 101% of these outcomes, with SHRs (95% CI) of 1.06 (1.01, 1.11) for stroke to 2.01 (1.45, 2.79) for hepatocellular carcinoma. For all-cause mortality and CVD, the relative risk was up to 39% higher in individuals with prediabetes compared with individuals within the normal glucose range. Of note, one meta-analysis did not detect an association between prediabetes classified according to IFG-ADA criteria and chronic kidney disease (low certainty of evidence). However, this meta-analysis applied a specific selection criterion and excluded studies on participants who developed diabetes after baseline [30], while in all other meta-analyses, including the other meta-analyses on chronic kidney disease [8], studies were only excluded when participants had diabetes at baseline.

Prediabetes was further associated with increased risk of sudden cardiac death, stomach and colorectal cancer combined, pancreatic cancer, Alzheimer’s dementia and vascular dementia (Fig. 2), but certainty of evidence was low or very low (ESM Table 7). There were no (or imprecise) associations between prediabetes and bronchus and lung cancer, prostate cancer, kidney cancer, depressive symptoms and cognitive impairment (low or very low certainty of evidence).

Figure 3 summarises the SHRs with the corresponding 95% CIs of each identified meta-analysis among specific patient groups. For patients with previous CVD, prediabetes (IFG-ADA) was associated with risk of all-cause mortality (low certainty of evidence) and with increased risk of further cardiovascular events (IFG-ADA, IGT, moderate certainty of evidence, and HbA_1c_, low certainty of evidence) (ESM Table 7). There was low or very low certainty of evidence that prediabetes was associated with increased risk of stroke and poor outcomes in patients with history of stroke/transient ischaemic attack, or with major adverse cardiac events in patients with previous percutaneous coronary intervention. For stroke mortality, the direction of the meta-analyses for both patient groups also pointed to an increased SHR, although the 95% CIs were imprecisely estimated.

The heterogeneity in the included meta-analyses varied, with *I*^2^ between 0 and 83%. The variance of the true effect sizes (τ^2^) ranged between 0 and 0.917 (ESM Table 4), but *p* values were mostly <0.05. For ten associations, the 95% prediction intervals excluded unity (outcomes, risk of all-cause mortality; cardiovascular events; CHD; stroke; heart failure; and pancreatic cancer). This result means that the true effect sizes in future studies can be expected to point in the same direction as in the meta-analyses presented here; however, for most of the associations, smaller or null findings could be possible in other samples.

For meta-analyses with more than ten studies, there was no indication of small study effects/publication bias for most of the associations (ESM Fig. 1), with the exception of the association between IFG-ADA and IGT regarding cardiovascular events and for IFG-WHO regarding CHD. For these meta-analyses, there was indication of small study effects according to Egger’s test (*p* < 0.1) and indication for missing of small studies in the funnel plots.

In general, there was indication for stronger associations for IGT than for IFG or HbA_1c_ regarding several outcomes (e.g. risk of all-cause mortality, cardiovascular events, CHD and stroke). However, the 95% CIs mostly overlapped, and thus, a clear gradient was observed only for risk of all-cause mortality.

## Discussion

Prediabetes was associated with a higher risk of all-cause mortality and incident cardiovascular events, CHD, stroke, heart failure, atrial fibrillation, chronic kidney disease, total cancer, total liver cancer, hepatocellular carcinoma, breast cancer and all-cause dementia, with moderate certainty of evidence, in meta-analyses including individuals from the general population and with worse prognosis in patients with previous cardiovascular problems. Summary effect estimates were higher for IGT-defined prediabetes and weaker for HbA_1c_-defined prediabetes compared with IFG-defined prediabetes; this was most pronounced for risk of all-cause mortality as an outcome. No associations were observed between prediabetes and incident depressive symptoms, cognitive impairment or some specific cancers. The risk of bias was high for most meta-analyses included in this umbrella review, showing that authors of future meta-analyses should adhere to established recommendations regarding the conduct and reporting of systematic reviews and meta-analyses and should assess and discuss the risk of bias of primary studies. No eligible meta-analyses could be identified for the incidence of neuropathy and retinopathy.

### All-cause mortality

Prediabetes defined by IFG or IGT, but not prediabetes defined using HbA_1c_, was associated with an 8–25% increased risk of all-cause mortality. In comparison, the risk for all-cause death was between 60 and 100% higher in people with diabetes than in people without diabetes in recent studies [35, 36]. The summary effect size was higher for prediabetes defined by IGT than for that defined by IFG (without overlapping 95% CIs between IGT and IFG/WHO) or for HbA_1c_-defined prediabetes, pointing towards the superior utility of the OGTT in the identification of high-risk individuals.

### Cardiovascular outcomes

Prediabetes defined by IFG, IGT and HbA_1c_ was also positively associated with risk of CV mortality and multiple CV outcomes such as incident CVD, CHD, stroke, heart failure and atrial fibrillation. The SHRs for prediabetes and risk of CV mortality ranged from 1.2 to 1.3. These data can be compared with CVD-related mortality data from the USA from 2000 to 2015 showing risk for people with vs without diabetes of 1.8–2.1 [35, 37]. Thus, the excess risk conferred by prediabetes appears to be less than one-third when compared with the risk related to diabetes. As the identified meta-analyses on stroke did not distinguish between ischaemic and haemorrhagic stroke [23, 29], we also restricted our analysis to stroke as a combined outcome. Potential differences in the associations between prediabetes and both stroke types need studying further.

### Microvascular outcomes

One meta-analysis on prediabetes and chronic kidney disease was available, with an SHR of 1.10 and 1.25 depending on the underlying definition of IFG. This result can be compared with an approximate threefold higher risk of chronic kidney disease in people with diabetes than in those without diabetes [38]. This suggests that prediabetes, at least when defined by IFG, may be a substantially weaker risk factor for chronic kidney disease than diabetes.

Our study protocol also included diabetic retinopathy, macular oedema, vitreous haemorrhage and diabetes-related blindness as well as peripheral and autonomic (poly)neuropathy, diabetic foot ulcers and amputations as outcomes of interest, but our systematic literature search did not identify any relevant meta-analyses. Given that the prevalence of retinopathy was initially used to refine threshold levels of fasting plasma glucose, 2 h plasma glucose and HbA_1c_ for the diagnosis of diabetes [39], it is not surprising that the association between prediabetes and retinopathy has been reported to be weak [40]. In contrast, evidence is accumulating for the presence of peripheral and cardiac autonomic neuropathy in people with prediabetes [41–44], so this field needs further prospective studies to quantify this relationship.

### Cancer

Depending on how prediabetes was defined, the SHR for the association between prediabetes and total cancer incidence ranged from 1.11 to 1.25. In comparison, total cancer incidence and cancer mortality were 1.1- to 1.4-fold higher in people with diabetes than in those without diabetes [45]. Associations with moderate certainty of evidence between prediabetes and site-specific cancers appeared stronger for total liver cancer (twofold higher) and hepatocellular carcinoma (SHR 1.44) compared with other cancers. An umbrella review on type 2 diabetes and cancer incidence found varying associations with site-specific cancers, but in line with our study, there was evidence for a link with incident liver and colorectal cancer [35, 45, 46]. Overall, effect estimates for prediabetes and diabetes appear similar with cancer as an outcome.

### Mental health

People with prediabetes showed an 18–47% increased risk for dementia (all-cause, Alzheimer’s or vascular) than people without prediabetes. In people with diabetes, the excess risk has been estimated to be 60–130% compared with people without diabetes, depending on the type of dementia and the sex of the individual [47]. In contrast, no associations were found between prediabetes and cognitive impairment or depressive symptoms, although increased risks for both conditions have been reported for people with diabetes [9, 28, 48]. It is possible that more pronounced hyperglycaemia is required for the development of depressive symptoms or clinical depression than that found in prediabetes, whereas the discrepancies of the data between dementia and cognitive impairment are more challenging to interpret. However, it should be noted that the meta-analyses were based on small numbers of primary studies, and future studies might add evidence to these findings.

### Comparison between different prediabetes definitions

In the meta-analyses included in this report, different definitions for prediabetes were used, and for some outcomes, meta-analyses for multiple definitions were conducted separately whenever possible. In general, the SHRs were in the same direction independently of the used prediabetes definition. A clear difference was seen for risk of all-cause mortality, CHD and stroke, with the strongest association for IGT-defined prediabetes and an intermediate effect size for IFG-defined prediabetes [7, 29]. For the other outcomes, overlapping 95% CIs of the different prediabetes definitions precluded any firm conclusions. Of note, all meta-analyses on prediabetes defined by HbA_1c_ included ≤5 primary studies, which might explain this lack of evidence. However, HbA_1c_ shows only relatively weak correlations with fasting and 2 h plasma glucose in people without type 2 diabetes, meaning that its main determinants in people without diabetes are not glucose-related and are linked to different underlying (patho)physiological processes [49].

### Risk of bias and certainty of evidence

Based on the ROBIS tool, all except four of the reports identified for this umbrella review were graded as being at high risk of bias. Major potential sources of bias comprised unclear prediabetes definitions and issues with search strategy, data extraction and/or assessment and discussion of the risk of bias of the primary studies. Although ROBIS is a validated Cochrane tool with which to evaluate the risk of bias in such studies, it is limited by the fact that it only distinguishes between high and low risk of bias. However, it has to be emphasised that authors of systematic reviews and meta-analyses should adhere to established guidelines regarding the conduct and reporting of their reviews. In addition, risk of bias evaluation of the primary studies is a crucial aspect in grading of the evidence and needs to be included in meta-analyses.

According to the GRADE tool, cohort studies start with a low certainty of evidence because of the nature of the observational study design, implying the possibility of residual or unknown confounding. We checked the confounders included in all primary studies, and in most of them, important known confounders were considered. Furthermore, in our umbrella review, we upgraded the certainty of evidence when a dose–response gradient was expected. Therefore, we assumed a dose–response gradient in the following situations: when associations were stronger for IFG-defined prediabetes when IFG was defined by the WHO compared with the ADA (different cut-off values); when associations were stronger for IGT- than for IFG-defined prediabetes; or when the original meta-analysis conducted a dose–response meta-analysis. However, we did not investigate dose–response meta-analyses by ourselves, since we investigated prediabetes as a binary variable (yes/no). Moreover, several of the associations in our report were graded as having a low or very low certainty of evidence, showing that more studies are needed to strengthen the evidence.

### Strengths and limitations

This umbrella review had several strengths. It provides the first systematic overview of the evidence from meta-analyses on the association between prediabetes and the incidence of ‘diabetes-related’ complications. We extracted data from primary studies and recalculated the SHR after excluding studies that were not in accordance with our a priori defined prediabetes categories. Whenever possible, we calculated effect estimates for all available prediabetes definitions. Risk of bias and certainty of evidence were assessed using the established ROBIS and GRADE tools, respectively. Finally, we identified gaps in the literature pointing towards future research needs.

Our umbrella review also had limitations, mostly related to the included meta-analyses or primary studies. First, the primary studies included in the identified meta-analyses had an observational design so that residual confounding cannot be ruled out. Second, study participants with prediabetes may have developed type 2 diabetes during the follow-up period, and this was usually not accounted for. Third, for most of the meta-analyses included in this umbrella review, risk of bias was high, and the certainty of evidence for some associations was low or very low, so that future high-quality studies are needed to corroborate our findings. Fourth, we did not explore heterogeneity in subgroups or sensitivity analyses, although whenever possible we differentiated between different prediabetes definitions. Finally, publication bias could not be assessed for most associations because of the small number of primary studies contributing to many of the identified meta-analyses.

### Conclusions

Prediabetes was associated with a higher relative risk of all-cause mortality and higher incidences of CV events, CHD, stroke, heart failure, atrial fibrillation, chronic kidney disease, total cancer, liver cancer, hepatocellular carcinoma, breast cancer and all-cause dementia with moderate certainty of evidence. Effect estimates were lower than comparable effect estimates for type 2 diabetes as exposure, suggesting a dose–response gradient in the relationship with complications. For risk of all-cause mortality, the association was stronger for IGT- than for IFG- or HbA_1c_-defined prediabetes, which points to towards the superior utility of the OGTT in the identification of high-risk individuals. Of note, no meta-analyses were found for associations between prediabetes and common diabetes-related complications such as neuropathy and retinopathy. The consistency of the results, the high global prevalence of prediabetes and the possibility of improving glucose metabolism and related risk factors in people with prediabetes using lifestyle interventions should lead to intensified research in this field. Given the identified research gaps and high risk of bias of the meta-analyses included in this umbrella review, higher-quality studies (both primary studies and meta-analyses) on the associations between prediabetes and multiple health outcomes are required to more robustly estimate the potential medical and cost-related benefits of intervention measures at this stage.

## Supplementary information

ESM (PDF 1.63 mb)

## Data Availability

All data generated or analysed during this study are included in this published article (and its supplementary information files).

## Abbreviations

IFG: Impaired fasting glucose
IGT: Impaired glucose tolerance
SHR: Summary HR
