# Supplementary material for: Prediabetes and risk of mortality, diabetes-related complications and comorbidities: umbrella review of meta-analyses of prospective studies
Source: Diabetologia. 2021 Oct 31;65(2):275–85. doi: 10.1007/s00125-021-05592-3 (PMC8741660; doi:10.1007/s00125-021-05592-3)
Supplement: Supplementary file 1 — (PDF 1.63 mb) [file 125_2021_5592_MOESM1_ESM.pdf]

Electronic supplementary material (ESM)

Prediabetes and risk of mortality, diabetes-related complications and comorbidities: umbrella review of meta-analyses of prospective studies

Sabrina Schlesinger[1,2], Manuela Neuenschwander[1,2], Janett Barbaresko[1], Alexander Lang[1], Haifa Maalmi[2,3], Wolfgang Rathmann[1,2], Michael Roden[2,3,4], Christian Herder[2,3,4]

[1]Institute for Biometrics and Epidemiology, German Diabetes Center (Deutsches Diabetes-Zentrum/DDZ), Leibniz Center for Diabetes Research at Heinrich Heine University Düsseldorf, Düsseldorf, Germany;

[2]German Center for Diabetes Research (DZD), Partner Düsseldorf, Germany;

[3]Institute for Clinical Diabetology, German Diabetes Center (Deutsches Diabetes-Zentrum/DDZ), Leibniz Center for Diabetes Research at Heinrich Heine University Düsseldorf, Düsseldorf, Germany;

[4]Department of Endocrinology and Diabetology, Medical Faculty and University Hospital, Heinrich-Heine University, Düsseldorf, Germany.

Corresponding author

Sabrina Schlesinger

E-mail: sabrina.schlesinger@ddz.de

ESM Table 1: Search strategy for e.g. PubMed

#1	(Prediabetic State[MeSH] or Glucose Intolerance[MeSH] OR Glycated Hemoglobin A [MeSH]) OR (prediabetes OR prediabetic OR “impaired glucose tolerance” OR IGT OR “impaired fasting glucose” OR IFG OR “impaired FPG”) OR (“HbA(1c)” OR HbA1 OR HbA1c OR “HbA 1c” OR ((glycosylated OR glycated) hemoglobin))
#2	"systematic review" OR meta-analysis
#3	Combine: #1 AND #2

ESM Table 2: Extracted data

From meta-analyses	• name of the first author<br>• publication year<br>• number of included studies<br>• study design of the primary studies<br>• definition of prediabetes<br>• outcome<br>• total number of cases and participants<br>• definition of the comparison<br>• statistical model that had been used to summarize the risk ratios<br>• summary risk ratios and their 95% confidence intervals<br>• confounders<br>• information on funding<br>• and conflict of interest
From primary studies	• name of the first author<br>• publication year<br>• country<br>• definition of prediabetes<br>• outcome<br>• number of total cases and participants (if not provided in the meta-analyses)<br>• risk estimates (hazard ratios, relative risks or odds ratios) that adjusted for the most confounders along with their 95% confidence intervals<br>• confounders (if not provided in the meta-analyses)

ESM Table 3: List of excluded studies

**Reasons for exclusion**	**Reference**
Not relevant exposure, including not relevant prediabetes definition	[1-39]
Not relevant population/in patients with diabetes	[40-51]
Not relevant data	[52-64]
No meta-analysis	[65-77]
Superseded	[78-85]
Conference abstract/editorial/comment	[86-93]
Not systematic	[94-97]
Protocol	[98-100]
Not relevant primary study design	[101-108]
Not relevant outcome	[109, 110]
Duplicate	[111-113]

ESM Table 4: Characteristics of the included meta-analyses on prediabetes and diabetes-related complications, comorbidities, and mortality

Outcome	Reference	*No.* of primary studies	Prediabetes definition	Prediabetes assessment criteria	No. of participants/ No. of cases[a]	Summary HR (95% CI)	I^2 (%)	τ^2	95% PI
**All-cause mortality**									
All-cause mortality	Cai 2020[114]	18	IFG	ADA	6’320’757 / na	**1.08 (1.03; 1.13)**	54	0.003	0.95; 1.23
All-cause mortality	Cai 2020[114]	19	IFG	WHO	325’446 / na	**1.13 (1.05; 1.20)**	41	0.007	0.93; 1.36
All-cause mortality	Cai 2020[114]	15	IGT	ADA/WHO	210’114 / na	**1.25 (1.17; 1.32)**	28	0.003	1.08; 1.44
All-cause mortality	Cai 2020[114]	7	HbA_{1c}	ADA	241’654 / na	0.98 (0.91; 1.05)	11	0.001	0.87; 1.11
All-cause mortality	Cai 2020[114]	2	IFG and/or IGT	ADA	2428 / na	1.14 (0.82; 1.58)	0	0.000	na
All-cause mortality	Cai 2020[114]	8	IFG and/or IGT	WHO	277’949 / na	**1.17 (1.13; 1.20)**	0	0.000	1.12; 1.21
All-cause mortality	Cai 2020[114]	4	IFG and/or HbA_{1c}	ADA	308’326 / na	1.05 (0.94; 1.16)	0	0.000	0.88; 1.24
Long-term all-cause mortality (in patients after PCI)	Li 2020[115]	3	HbA_{1c}	ADA	4085 / 298	1.18 (0.92; 1.50)	0	0.000	0.24; 5.72
Short-term all-cause mortality (in patients after PCI)	Li 2020[115]	2	HbA_{1c}	ADA	3633 / 122	1.00 (0.69; 1.44)	0	0.000	na
All-cause mortality (in patients with atherosclerotic cardiovascular disease)	Cai 2020[114]	5	IFG	ADA	6633 / na	1.60 (1.15; 2.22)	70	0.091	0.54; 4.78
All-cause mortality (in patients with atherosclerotic cardiovascular disease)	Cai 2020[114]	5	IFG	WHO	29497 / 6492	1.19 (0.98; 1.45)	65	0.023	0.67; 2.12
All-cause mortality (in patients with atherosclerotic cardiovascular disease)	Cai 2020[114]	3	IGT	ADA/WHO	1919 / 140	1.34 (0.94; 1.93)	0	0.000	0.13; 14.00

All-cause mortality (in patients with atherosclerotic cardiovascular disease)	Cai 2020[114]	2	HbA_{1c}	ADA	3205 / 137	2.30 (0.56; 9.41)	77	0.814	na
All-cause mortality (in patients with atherosclerotic cardiovascular disease)	Cai 2020[114]	2	IFG and/or IGT	ADA	1067 / na	1.62 (0.96; 2.73)	0	0.000	na
All-cause mortality (in patients with atherosclerotic cardiovascular disease)	Cai 2020[114]	2	IFG and/or IGT	WHO	2406 / na	1.07 (0.75; 1.52)	0	0.000	na
**Cardiovascular outcomes & cardiovascular mortality**									
CV events	Cai 2020[114]	22	IFG	ADA	1’190’425 / na	**1.09 (1.03; 1.15)**	61	0.008	0.90; 1.32
CV events	Cai 2020[114]	25	IFG	WHO	344’915 / na	**1.20 (1.09; 1.34)**	60	0.032	0.82; 1.77
CV events	Cai 2020[114]	19	IGT	ADA/WHO	223’370 / na	**1.23 (1.13; 1.34)**	44	0.012	0.96; 1.58
CV events	Cai 2020[114]	8	HbA_{1c}	ADA	255’198 / na	1.05 (0.97; 1.13)	42	0.005	0.86; 1.27
CV events	Cai 2020[114]	2	IFG and/or IGT	ADA	2760 / na	1.15 (0.91; 1.45)	0	0.000	na
CV events	Cai 2020[114]	7	IFG and/or IGT	WHO	276’787 / na	1.10 (0.99; 1.21)	25	0.004	0.89; 1.35
CV events	Cai 2020[114]	6	IFG and/or HbA_{1c}	ADA	319’644 / na	1.05 (0.97; 1.13)	0	0.000	0.94; 1.16
CV events	Cai 2020[114]	2	IFG and/or HbA_{1c} and/or IGT	ADA	86’808 / na	0.98 (0.92; 1.05)	0	0.000	na
CV events (in patients with atherosclerotic cardiovascular disease)	Cai 2020[114]	6	IFG	ADA	9370 / na	**1.33 (1.02; 1.75)**	81	0.085	0.54; 3.28
CV events (in patients with atherosclerotic cardiovascular disease)	Cai 2020[114]	5	IFG	WHO	16558 / na	1.49 (0.99; 2.24)	83	0.141	0.38; 5.82

CV events (in patients with atherosclerotic cardiovascular disease)	Cai 2020[114]	6	IGT	ADA/WHO	6369 / na	**1.52 (1.24; 1.85)**	0	0.000	1.15; 2.01
CV events (in patients with atherosclerotic cardiovascular disease)	Cai 2020[114]	4	HbA_{1c}	ADA	10990 / na	**1.24 (1.05; 1.48)**	0	0.000	0.85; 1.81
CV events (in patients with atherosclerotic cardiovascular disease)	Cai 2020[114]	2	IFG and/or HbA_{1c}	ADA	7153 / 209	**1.61 (1.07; 2.43)**	2	0.002	na
CV events (in patients with atherosclerotic cardiovascular disease)	Cai 2020[114]	2	IFG and/or HbA_{1c} and/or IGT	ADA	3455 / na	1.16 (0.86; 1.57)	0	0.000	na
CV mortality[c]	Huang 2016[116]	6	IFG	ADA	na / na	**1.27 (1.02; 1.58)**	na	na	na
CV mortality[c]	Huang 2016[116]	13	IFG	WHO	na / na	**1.20 (1.05; 1.38)**	na	na	na
CV mortality[c]	Huang 2016[116]	9	IGT	ADA/WHO	na / na	**1.30 (1.18; 1.44)**	na	na	na
CVD incidence[c]	Huang 2016[116]	9	IFG	ADA	na / na	**1.10 (1.03, 1.18)**	na	na	na
CVD incidence[c]	Huang 2016[116]	5	IFG	WHO	na / na	**1.39 (1.15, 1.68)**	na	na	na
CVD incidence[c]	Huang 2016[116]	4	IGT	ADA/WHO	na / na	**1.29 (1.11, 1.50)**	na	na	na
CHD	Cai 2020[114]	22	IFG	ADA	1’207’240 / na	**1.09 (1.05; 1.13)**	4	0.000	1.03; 1.15
CHD	Cai 2020[114]	12	IFG	WHO	86’407 / na	**1.17 (1.09; 1.26)**	0	0.000	1.08; 1.28
CHD	Cai 2020[114]	11	IGT	ADA/WHO	50’506 / na	**1.21 (1.09; 1.34)**	0	0.000	1.07; 1.36
CHD	Cai 2020[114]	3	HbA_{1c}	ADA	81’949 / na	**1.30 (1.04; 1.62)**	76	0.028	0.10; 16.90
CHD	Cai 2020[114]	5	IFG and/or IGT	WHO	50’217 / na	**1.17 (1.02; 1.35)**	0	0.000	0.94; 1.47
CHD	Cai 2020[114]	2	IFG and/or HbA_{1c}	ADA	73’987 / na	1.11 (0.88; 1.39)	0	0.000	na

CHD (in patients with atherosclerotic cardiovascular disease)	Cai 2020[114]	2	IFG	ADA	2967 / 536	1.10 (0.92; 1.30)	0	0.000	na
CHD (in patients with atherosclerotic cardiovascular disease)	Cai 2020[114]	2	IFG	WHO	11829 / 650	1.24 (0.99; 1.56)	0	0.000	na
CHD (in patients with atherosclerotic cardiovascular disease)	Cai 2020[114]	3	IGT	ADA/WHO	2545 / 209	1.14 (0.84; 1.54)	0	0.000	0.16; 8.10
CHD (in patients with atherosclerotic cardiovascular disease)	Cai 2020[114]	2	HbA_{1c}	ADA	6782 / 91	1.16 (0.65; 2.05)	0	0.001	na
Stroke	Cai 2020[114]	16	IFG	ADA	1’635’506 / na	**1.06 (1.01; 1.11)**	16	0.001	0.97; 1.16
Stroke	Cai 2020[114]	8	IFG	WHO	698’478 / na	**1.18 (1.10; 1.26)**	0	0.000	1.08; 1.28
Stroke	Cai 2020[114]	8	IGT	ADA/WHO	31’047 / na	**1.30 (1.10; 1.54)**	42	0.022	0.86; 1.98
Stroke	Mitsios 2018[117]	4	HbA_{1c}	ADA	44’431 / 1578	1.19 (0.87; 1.63)	61.9	0.054	0.35; 4.00
Stroke	Cai 2020[114]	2	IFG and/or IGT	WHO	3013 / na	1.16 (0.81; 1.65)	0	0.000	na
Stroke	Cai 2020[114]	2	IFG and/or HbA_{1c}	ADA	73’987 / na	1.01 (0.79; 1.30)	0	0.000	na
Stroke (in patients with atherosclerotic cardiovascular disease)	Cai 2020[114]	2	IFG	ADA	2967 / 287	0.99 (0.63; 1.54)	62	0.068	na
Atrial fibrillation	Aune 2018[118]	3	IFG	ADA/ WHO	248’598 / 3301	**1.13 (1.003; 1.27)**	0	0.000	0.52; 2.45
Atrial fibrillation	Aune 2018[118]	3	IFG	ADA	248’598 / 3301	**1.13 (1.03; 1.24)**	0	0.000	0.63; 2.04
Heart failure	Cai 2021	10	IFG	ADA	8’962’851 / na	**1.10 (1.06, 1.14)**	55	0.001	1.01; 1.20
Heart failure	Cai 2021	6	IFG	WHO	22941 / na	**1.18 (1.07, 1.30)**	0	0.000	1.3; 1.36
Heart failure	Cai 2021	3	IGT	ADA/ WHO	2317 / 271	**1.58 (1.04, 2.39)**	26	0.042	0.04; 65.80

Sudden cardiac death	Aune 2018[119]	2	IFG / IGT	ADA	7766 / 215	**1.52 (1.08; 2.14)**	0	0.000	na
Stroke (in patients with history of stroke/TIA)	Pan 2019[112]	3	IFG / IGT	ADA /WHO	8865 / na	1.45 (0.98; 2.14)	42	0.051	0.03; 65.75
Stroke (in patients with history of stroke/TIA)	Pan 2019[112]	2	IFG	WHO	6022 / na	1.17 (0.55; 2.48)	64.6	0.203	na
Poor outcome of stroke (in patients with history of stroke/TIA)[b]	Pan 2019[112]	5	IFG / IGT/ HbA_{1c}	ADA /WHO	7045 / na	**1.41 (1.01; 1.97)**	56	0.044	0.50; 3.95
Poor outcome of stroke (in patients with history of stroke/TIA)[b]	Pan 2019[112]	2	IFG	WHO	6022 / na	**1.41 (1.05; 1.90)**	29.9	0.014	na
Stroke mortality (in patients with history of stroke/TIA)	Pan 2019[112]	4	IFG/IGT	ADA/ WHO	6850 / na	1.40 (0.68; 2.91)	65	0.340	0.07; 27.52
Stroke mortality (in patients with history of stroke/TIA)	Pan 2019[112]	2	IFG	WHO	6022 / na	1.64 (0.67; 4.01)	37.5	0.180	na
Stroke mortality (in patients with history of stroke/TIA)	Pan 2019[112]	2	HbA_{1c}	ADA	828 / na	1.28 (0.30; 5.51)	81.4	0.917	na
MACE (in patients after PCI)	Zhao 2020[120]	10	IFG/IGT/ HbA_{1c}	ADA/WHO	6272 / na	**1.41 (1.14; 1.75)**	30.8	0.035	0.86; 2.32
MACE (in patients after PCI)	Zhao 2020[120]	2	IGT	ADA/WHO	461 / na	**1.62 (1.07; 2.46)**	0	0.000	na
MACE (in patients after PCI)	Zhao 2020[120]	3	HbA1c	ADA	3352 / na	1.38 (0.88; 2.17)	63	0.099	0.01; 196.94
**Microvascular outcomes**									
Chronic kidney disease	Echouffo-Tcheugui 2016[121]	8	IFG	ADA/WHO	170’081 / 15’259	**1.10 (1.01; 1.20)**	80.4	0.007	0.88; 1.39
Chronic kidney disease	Echouffo-Tcheugui 2016[121]	6	IFG	WHO	57’759 / 2445	**1.25 (1.02; 1.53)**	83.2	0.048	0.63; 2.45
Chronic kidney disease	Mutie 2020[122]	4	IFG	ADA	137’483 / na	0.97 (0.94; 1.01)	0	0.000	0.89; 1.06
Chronic kidney disease	Mutie 2020[122]	3	HbA_{1c}	ADA	19834 / na	1.07 (0.94; 1.21)	0	0.000	0.48; 2.38

**Cancer**									
Total cancer	Huang 2014[123]	5	IFG	ADA/WHO	87’916 / na	1.13 (1.00; 1.28)	20.8	0.004	0.85; 1.51
Total cancer	Huang 2014[123]	4	IFG	WHO	85’478 / na	**1.11 (1.01; 1.22)**	0	0.000	0.90; 1.37
Total cancer	Huang 2014[123]	6	IGT	ADA/WHO	82’296 / na	**1.25 (1.02; 1.53)**	39.5	0.024	0.74; 2.09
Total cancer	Huang 2014[123]	2	IFG / IGT	WHO	46’121 / na	**1.11 (1.02; 1.21)**	0	0.000	na
Stomach/colorectal cancer	Huang 2014[123]	3	IFG / IGT	ADA/ WHO	52’113 / na	**1.55 (1.15; 2.09)**	59.7	0.041	0.06; 39.06
Liver cancer	Huang 2014[123]	3	IFG / IGT	ADA/WHO	53’971 / na	**2.01 (1.45; 2.79)**	0	0.000	0.24; 16.69
Hepatocellular carcinoma	Xu 2017[124]	5	IFG / IGT	ADA/WHO	1’366’784 / na	**1.44 (1.09; 1.90)**	40.8	0.038	0.67; 3.10
Hepatocellular carcinoma	Xu 2017[124]	3	IFG	ADA	1’303’726 / na	**1.23 (1.03; 1.47)**	14.8	0.009	0.23; 6.47
Bronchus and lung cancer	Huang 2014[123]	2	IFG / IGT	ADA/WHO	47’093 / na	1.35 (0.86; 2.11)	74.0	0.079	na
Prostate cancer	Huang 2014[123]	3	IFG / IGT	ADA/ WHO	104’426 / na	1.19 (0.86; 1.65)	48.4	0.040	0.04; 32.97
Kidney or bladder cancer	Huang 2014[123]	2	IFG / IGT	ADA/ WHO	99’406 / na	0.80 (0.55; 1.16)	0	0.000	na
Breast cancer	Huang 2014[123]	4	IFG / IGT	ADA/WHO	288’306 / na	**1.19 (1.03; 1.38)**	0	0.000	0.86; 1.64
Breast cancer	Huang 2014[123]	2	IFG	WHO	235’757 / na	1.13 (0.95; 1.35)	0	0.000	na
Pancreatic cancer	Fu 2016[125]	5	IFG / IGT	ADA/WHO	1’809’891 / na	**1.22 (1.11; 1.34)**	0	0.000	1.05; 1.35
Pancreatic cancer	Fu 2016[125]	3	IFG	ADA	1’747’230 / na	**1.25 (1.12, 1.39)**	17.6	0.002	0.50; 3.10
**Mental/cognitive outcomes**									
Depressive symptoms	Tong 2016[126]	4	IFG/IGT/ HbA_{1c}	ADA/WHO	14’660 / na	1.07 (0.80; 1.43)	73.1	0.056	0.32; 3.53
Depressive symptoms	Tong 2016[126]	2	IFG	ADA	10’128 / na	0.91 (0.67; 1.23)	70.2	0.033	na
All-cause dementia[c]	Xue 2019[113]	9	IFG/IGT	na	na / na	**1.18 (1.02; 1.36)**	22	na	na
All-cause dementia[c]	Xue 2019[113]	?	IFG	5.6-6.9	na / na	**1.27 (1.08; 1.49)**	0	na	na

All-cause dementia[c]	Xue 2019[113]	?	IGT	na	na / na	**1.40 (1.03; 1.91)**		na	na
Alzheimer's dementia[c]	Xue 2019[113]	5	IFG / IGT	na	na / na	**1.36 (1.09; 1.70)**	14.0	na	na
Vascular dementia[c]	Xue 2019[113]	3	IFG / IGT	na	na / na	**1.47 (1.01; 2.15)**	0	na	na
Cognitive impairment[c]	Xue 2019[113]	5	IFG / IGT	na	na / na	0.96 (0.85; 1.09)	0	na	na

na, not available; PI, prediction interval.

[a] total number of participants / cases as extracted from the meta-analyses, might include individuals with diabetes, because number of participants / cases was not always available for the prediabetes subgroup only
[b] poor outcomes defined as degree of disability or dependence in the daily activities of people who have suffered a stroke or other causes of neurological disability
[c] could not be recalculated because of missing information.

Boldface shows summary associations with precise 95% CIs, excluding the null value.

ESM Table 5: Important confounders considered in each primary study included in the meta-analyses

	Confounders											
	Age	Sex	Ethnicity[a]	Education/income	BMI/ waist	Smoking	Physical activity	Alcohol intake	Blood pressure/ treatment	Blood lipids/ treatment	Chronic diseases	Further confounders
**All-cause mortality: IFG-ADA**												
Yeboah, 2011	✓	✓	✓		✓	✓			✓	✓		
Deedwania, 2013	✓	✓	✓	✓	✓	✓	✓	✓	✓	✓	✓	ankle arm index ratio, haemoglobin, albumin, uric acid, serum insulin, LV hypertrophy, atrial fibrillation, bundle branch block, CRP
Samaras, 2015	✓	✓			✓	✓			✓			
Jin, 2008	✓	✓			✓				✓	✓	✓	
Laukkanen, 2013	✓	✓			✓	✓		✓	✓	✓	✓	family history of CHD
Kim, 2016	✓	✓			✓	✓			✓	✓		family history of CVD
Rijkelijkhuizen, 2007	✓	✓										
Tang, 2019	✓	✓	✓	✓								family history of diabetes
Lu, 2019	✓	✓		✓	✓	✓	✓	✓	✓	✓		family history of diabetes
Wen, 2005	✓	✓			✓	✓			✓	✓		
de Abreu, 2017	✓	✓				✓						
Parizadeh, 2019	✓			✓	✓	✓			✓	✓	✓	family history of diabetes, eGFR
Rhee, 2020	✓	✓			✓	✓	✓	✓	✓	✓	✓	
Jiang, 2020	✓	✓		✓	✓	✓			✓	✓	✓	
Lazo-Porras, 2020	✓	✓		✓	✓	✓	✓	✓	✓	✓		population group
Kim, 2017	✓	✓		✓	✓	✓	✓	✓	✓	✓		antithrombotics
Mongraw-Chaffin, 2017	✓	✓	✓	✓	✓	✓			✓	✓		diabetes treatment

	Confounders											
	**Age**	**Sex**	**Ethnicity[a]**	**Education/income**	**BMI/ waist**	**Smoking**	**Physical activity**	**Alcohol intake**	**Blood pressure/ treatment**	**Blood lipids/ treatment**	**Chronic diseases**	**Further confounders**
Shi, 2016	✓	✓		✓	✓	✓	✓	✓	✓			intake of energy, fat and fibre
**All-cause mortality: IFG-WHO**												
Lu, 2003	✓	✓			✓	✓	✓	✓	✓	✓		study center
Nakagami, 2004	✓	✓			✓	✓			✓	✓		cohort
Hunt, 2004	✓	✓	✓									
Magliano, 2010		✓		✓	✓	✓			✓	✓	✓	
Saydah, 2001	✓	✓	✓	✓	✓	✓	✓		✓	✓		
DECODE, 2001	✓	✓			✓	✓			✓	✓		center
Rodriguez, 2002	✓	✓			✓		✓		✓	✓		fibrinogen
Tsai, 2008	✓	✓				✓						
Wild, 2005	✓	✓				✓			✓	✓	✓	
Samaras, 2015	✓	✓			✓	✓			✓			
Henry, 2002	✓	✓			✓	✓			✓	✓		
Barr, 2007	✓	✓			✓	✓			✓	✓	✓	
Rijkelijkhuizen, 2007	✓	✓										
Wändell, 2005	✓	✓			✓				✓			
Parizadeh, 2019	✓			✓	✓	✓			✓	✓	✓	family history of diabetes, eGFR
Lu, 2019	✓	✓		✓	✓	✓	✓	✓	✓	✓		family history of diabetes
Jiang, 2020	✓	✓		✓	✓	✓			✓	✓	✓	

	Confounders											
	**Age**	**Sex**	**Ethnicity[a]**	**Education/income**	**BMI/ waist**	**Smoking**	**Physical activity**	**Alcohol intake**	**Blood pressure/ treatment**	**Blood lipids/ treatment**	**Chronic diseases**	**Further confounders**
Warren, 2017	✓	✓	✓	✓	✓	✓		✓	✓	✓		family history of diabetes, eGFR
Wen, 2005	✓	✓			✓	✓			✓	✓		
**All-cause mortality: IGT- ADA/WHO**												
Saydah, 2001	✓	✓	✓	✓	✓	✓	✓		✓	✓		
Hiltunen, 2005	✓	✓			✓	✓	✓		✓		✓	self-perceived health
Rodriguez, 2002	✓	✓			✓		✓		✓	✓		fibrinogen
Wild, 2005	✓	✓				✓			✓	✓	✓	
Stengard, 1992	✓	✓			✓	✓			✓	✓		functional capacity
Magliano, 2010		✓		✓	✓	✓			✓	✓	✓	
Nakagami, 2004	✓	✓			✓	✓			✓	✓		cohort
Barr, 2007	✓	✓			✓	✓			✓	✓	✓	
DECODE, 2001	✓	✓			✓	✓			✓	✓		center
Kokubo, 2010	✓	✓			✓	✓		✓	✓	✓		
Lu, 2019	✓	✓		✓	✓	✓	✓	✓	✓	✓		family history of diabetes
Jiang, 2020	✓	✓		✓	✓	✓			✓	✓	✓	
Warren, 2017	✓	✓	✓	✓	✓	✓		✓	✓	✓		family history of diabetes, eGFR
Parizadeh, 2019	✓			✓	✓	✓			✓	✓	✓	family history of diabetes, eGFR
Fang, 2019	✓	✓			✓	✓			✓	✓	✓	insulin resistance, eGFR
**All-cause mortality: HbA1c-ADA**												

	Confounders											
	Age	Sex	Ethnicity[a]	Education/income	BMI/ waist	Smoking	Physical activity	Alcohol intake	Blood pressure/ treatment	Blood lipids/ treatment	Chronic diseases	Further confounders
Gordon-Dseagu, 2015	✓	✓		✓	✓	✓						
Paprott, 2015	✓	✓		✓	✓	✓	✓	✓	✓	✓	✓	
Kim, 2016	✓	✓			✓	✓			✓	✓		family history of CVD
Jiang, 2020	✓	✓		✓	✓	✓			✓	✓	✓	
Lu, 2019	✓	✓		✓	✓	✓	✓	✓	✓	✓		family history of diabetes
Tang, 2019	✓	✓	✓	✓								family history of diabetes
Lazo-Porras, 2020	✓	✓		✓	✓	✓	✓	✓	✓	✓		population group
**All-cause mortality: IGT/IFG - ADA**												
Hadaegh, 2015	✓	✓			✓	✓			✓	✓		
Valdes, 2009	✓	✓			✓	✓			✓	✓	✓	
**All-cause mortality: IGT/IFG - WHO**												
Saydah, 2001	✓	✓	✓	✓	✓	✓	✓		✓	✓		
Rodriguez, 2002	✓	✓			✓		✓		✓	✓		fibrinogen
Kowall, 2011	✓	✓			✓	✓	✓	✓	✓	✓	✓	parental diabetes
Evans, 2015	✓	✓		✓								
Hu, 2003	✓	✓			✓	✓			✓	✓		center
Nakagami, 2004	✓	✓			✓	✓			✓	✓		cohort
Ma, 2003	✓	✓		✓	✓	✓		✓	✓			
Valdes, 2009	✓	✓			✓	✓			✓	✓	✓	

	Confounders											
	**Age**	**Sex**	**Ethnicity[a]**	**Education/income**	**BMI/ waist**	**Smoking**	**Physical activity**	**Alcohol intake**	**Blood pressure/ treatment**	**Blood lipids/ treatment**	**Chronic diseases**	**Further confounders**
**All-cause mortality: IGF/HbA1c-ADA**												
Tang, 2019	✓	✓	✓	✓								family history of diabetes
Hubbard, 2019	✓	✓		✓	✓	✓	✓			✓	✓	aspirin use
Rhee, 2016	✓	✓			✓	✓	✓	✓	✓		✓	study center, year of screening examination, family history of diabetes
Kim, 2016	✓	✓			✓	✓			✓	✓		family history of CVD
**Long-term all-cause mortality (in patients after PCI): HbA1c-ADA**												
Naito, 2014	✓				✓					✓		LVEF, haemoglobin, eGFR, multivessel disease
Shin, 2016	✓	✓			✓					✓		LVEF, Killip class, troponin I
Aggarwal, 2016	✓	✓										
**Short-term all-cause mortality (in patients after PCI): HbA1c-ADA**												
Aggarwal, 2016	✓	✓										
Shin, 2016	✓	✓			✓					✓		LVEF, Killip class, troponin I
**All-cause mortality (in patients with atherosclerotic cardiovascular disease): IFG-ADA**												
Janszky, 2009	✓	✓		✓	✓		✓		✓	✓	✓	apo B ⁄ apo A ratio, Q wave infarction and education
Silbernagel, 2011	✓	✓			✓	✓			✓	✓	✓	eGFR, Friesinger score, glycated hemoglobin
Ding, 2014	✓	✓		✓	✓	✓	✓	✓	✓	✓	✓	marriage, glomerular filtration rate, antiplatelet drugs
Slezak, 2018	✓	✓			✓	✓			✓	✓		survey
Fefer, 2008	✓	✓									✓	Killip class, LVEF
**All-cause mortality (in patients with atherosclerotic cardiovascular disease): IFG-WHO**												
Fisman, 2001	✓	✓			✓	✓				✓	✓	functional class, peripheral vascular disease, anginal syndrome

	Confounders											
	Age	Sex	Ethnicity[a]	Education/income	BMI/ waist	Smoking	Physical activity	Alcohol intake	Blood pressure/ treatment	Blood lipids/ treatment	Chronic diseases	Further confounders
Muhlestein, 2003	✓	✓				✓			✓	✓	✓	type of PCI, LVEF, family history, glycemic status
Nigam, 2007	✓	✓			✓	✓			✓	✓	✓	
Giraldez, 2013	✓	✓			✓	✓			✓		✓	ST-segment changes on qualifying event ECG, white blood cell count, Killip class, creatinine clearance, heart rate, positive baseline troponin,
Kiviniemi, 2019	✓	✓			✓				✓			grading for angina pectoris, SYNTAX Score, and LVEF
**All-cause mortality (in patients with atherosclerotic cardiovascular disease): IGT-ADA/WHO**												
Ding, 2014	✓	✓		✓	✓	✓	✓	✓	✓	✓	✓	marriage, glomerular filtration rate, antiplatelet drugs
George, 2015	✓	✓				✓			✓	✓	✓	discharge diagnosis
Kiviniemi, 2019	✓	✓			✓				✓			grading for angina pectoris, SYNTAX Score, and LVEF
**All-cause mortality (in patients with atherosclerotic cardiovascular disease): HbA1c-ADA**												
Shin, 2016	✓	✓			✓	✓			✓	✓	✓	Killip class, LVEF, peak troponin I, serum creatinine, peak creatine kinase 2 isoenzyme, treated vessel, lesion type, PCI, CRP
Choi, 2018	✓	✓				✓			✓	✓	✓	bifurcation Lesion
**All-cause mortality (in patients with atherosclerotic cardiovascular disease): IFG/IGT-ADA**												
Hofsten, 2009	✓	✓									✓	Killip class II, LVEF, E/e=, EDT 140 ms, left atrial volume index>32 ml/m², NT-proBNP
Ding, 2014	✓	✓		✓	✓	✓	✓	✓	✓	✓	✓	marriage, glomerular filtration rate, antiplatelet drugs
**All-cause mortality (in patients with atherosclerotic cardiovascular disease): IFG/IGT-WHO**												
Lenzen, 2006	✓	✓								✓	✓	
Pararajasingam, 2016	✓	✓			✓	✓			✓	✓	✓	wall motion score index, PCI at admission, CABG at admission, PCI and CABG at admission, only reperfusion therapy at admission, CABG during follow-up, PCI during follow-up

	Confounders											
	**Age**	**Sex**	**Ethnicity[a]**	**Education/income**	**BMI/ waist**	**Smoking**	**Physical activity**	**Alcohol intake**	**Blood pressure/ treatment**	**Blood lipids/ treatment**	**Chronic diseases**	**Further confounders**
Pararajasingam, 2019	✓	✓										type of MI
**CV events: IFG-ADA**												
Khang, 2010	✓	✓			✓				✓	✓		
Deedwania, 2013	✓	✓	✓	✓	✓	✓	✓	✓	✓	✓	✓	ankle arm index ratio, haemoglobin, albumin, uric acid, serum insulin, LV hypertrophy, atrial fibrillation, bundle branch block, CRP
Kim, 2013	✓				✓	✓			✓	✓		family history of CVD
Ma, 2012	✓	✓		✓	✓			✓	✓			
Kim, 2016	✓	✓			✓	✓			✓	✓		family history of CVD
Schöttker, 2013	✓	✓				✓			✓	✓		
Yeboah, 2011	✓	✓	✓		✓	✓			✓	✓		
Levitzky, 2008	✓	✓			✓	✓			✓	✓		
Kokubo, 2010	✓	✓			✓	✓		✓	✓	✓		
Wang, 2007a	✓	✓				✓	✓	✓		✓	✓	
Liu, 2007	✓	✓				✓				✓		CVD family history
Rijkelijkhuizen, 2007	✓	✓										
Laukkanen, 2013	✓	✓			✓	✓		✓	✓	✓	✓	family history of CHD
Jin, 2008	✓	✓			✓				✓	✓	✓	
Tang, 2019	✓	✓	✓	✓								family history of diabetes
Vistisen, 2018	✓	✓	✓			✓			✓	✓	✓	
Lu, 2019	✓	✓		✓	✓	✓	✓	✓	✓	✓		family history of diabetes

	Confounders											
	Age	Sex	Ethnicity[a]	Education/income	BMI/ waist	Smoking	Physical activity	Alcohol intake	Blood pressure/ treatment	Blood lipids/ treatment	Chronic diseases	Further confounders
Kim, 2017	✓	✓		✓	✓	✓	✓	✓	✓	✓		antithrombotics
Jiang, 2020	✓	✓		✓	✓	✓			✓	✓	✓	
Mongraw-Chaffin, 2017	✓	✓	✓	✓	✓	✓			✓	✓		diabetes treatment
Wen, 2005	✓	✓			✓	✓			✓	✓		
Shi, 2016	✓	✓		✓	✓	✓	✓	✓	✓			intake of energy, fat and fibre
**CV events: IFG-WHO**												
Saydah, 2001	✓	✓	✓	✓	✓	✓	✓		✓	✓		
Lu, 2003	✓	✓			✓	✓	✓	✓	✓	✓		study center
Rodriguez, 2002	✓	✓			✓		✓		✓	✓		fibrinogen
Oizumi, 2008	✓	✓							✓			
Nakagami, 2004	✓	✓			✓	✓			✓	✓		cohort
Magliano, 2010		✓		✓	✓	✓			✓	✓	✓	
Wild, 2005	✓	✓				✓			✓	✓	✓	
DECODE, 2001	✓	✓			✓	✓			✓	✓		center
Nakanishi, 2004	✓	✓			✓	✓		✓	✓	✓		family history of T2D
Wang, 2007a	✓	✓				✓	✓	✓		✓	✓	
Barzilay, 1999	✓	✓	✓		✓	✓			✓	✓		
Nilsson, 2007	✓	✓										
Tsai, 2008	✓	✓				✓						

	Confounders											
	**Age**	**Sex**	**Ethnicity[a]**	**Education/income**	**BMI/ waist**	**Smoking**	**Physical activity**	**Alcohol intake**	**Blood pressure/ treatment**	**Blood lipids/ treatment**	**Chronic diseases**	**Further confounders**
Henry, 2002	✓	✓			✓	✓			✓	✓		
Hunt, 2004	✓	✓	✓									
Chien, 2008	✓	✓		✓	✓	✓	✓	✓				family history of CHD
Rijkelijkhuizen, 2007	✓	✓										
Barr, 2007	✓	✓			✓	✓			✓	✓	✓	
Wändell, 2005	✓	✓			✓				✓			
Vistisen, 2018	✓	✓	✓			✓			✓	✓	✓	
Warren, 2017	✓	✓	✓	✓	✓	✓		✓	✓	✓		family history of diabetes, eGFR
Lu, 2019	✓	✓		✓	✓	✓	✓	✓	✓	✓		family history of diabetes
Jiang, 2020	✓	✓		✓	✓	✓			✓	✓	✓	
Wen, 2005	✓	✓			✓	✓			✓	✓		
Onat, 2005	✓	✓				✓	✓					
**CV events: IGT-WHO/ADA**												
Saydah, 2001	✓	✓	✓	✓	✓	✓	✓		✓	✓		
Rodriguez, 2002	✓	✓			✓		✓		✓	✓		fibrinogen
Stengard, 1992	✓	✓			✓	✓			✓	✓		functional capacity
Wild, 2005	✓	✓				✓			✓	✓	✓	
Barr, 2007	✓	✓			✓	✓			✓	✓	✓	
Chien, 2008	✓	✓		✓	✓	✓	✓	✓				family history of CHD

	Confounders											
	**Age**	**Sex**	**Ethnicity[a]**	**Education/income**	**BMI/ waist**	**Smoking**	**Physical activity**	**Alcohol intake**	**Blood pressure/ treatment**	**Blood lipids/ treatment**	**Chronic diseases**	**Further confounders**
Barzilay, 1999	✓	✓	✓		✓	✓			✓	✓		
Magliano, 2010		✓		✓	✓	✓			✓	✓	✓	
Nakagami, 2004	✓	✓			✓	✓			✓	✓		cohort
DECODE, 2001	✓	✓			✓	✓			✓	✓		center
Oizumi, 2008	✓	✓							✓			
Wang, 2007a	✓	✓				✓	✓	✓		✓	✓	
Tai, 2004	✓	✓	✓									
Vistisen, 2018	✓	✓	✓			✓			✓	✓	✓	
Warren, 2017	✓	✓	✓	✓	✓	✓		✓	✓	✓		family history of diabetes, eGFR
Lu, 2019	✓	✓		✓	✓	✓	✓	✓	✓	✓		family history of diabetes
Jiang, 2020	✓	✓		✓	✓	✓			✓	✓	✓	
Cederberg, 2010		✓			✓	✓			✓	✓		family history of diabetes
Fang, 2019	✓	✓			✓	✓			✓	✓	✓	insulin resistance, eGFR
**CV events: HbA1c-ADA**												
Schöttker, 2013	✓	✓				✓			✓	✓		
Gordon-Dseagu, 2015	✓	✓		✓	✓	✓						
Kim, 2016	✓	✓			✓	✓			✓	✓		family history of CVD
Eastwood, 2015	✓	✓			✓	✓			✓	✓		
Jiang, 2020	✓	✓		✓	✓	✓			✓	✓	✓	

	Confounders											
	Age	Sex	Ethnicity[a]	Education/income	BMI/ waist	Smoking	Physical activity	Alcohol intake	Blood pressure/ treatment	Blood lipids/ treatment	Chronic diseases	Further confounders
Lu, 2019	✓	✓		✓	✓	✓	✓	✓	✓	✓		family history of diabetes
Tang, 2019	✓	✓	✓	✓								family history of diabetes
Vistisen, 2018	✓	✓	✓			✓			✓	✓	✓	
**CV events: IFG/IGT-ADA**												
Hadaegh, 2015	✓	✓			✓	✓			✓	✓		
Tian, 2018	✓	✓			✓	✓	✓	✓	✓			family history of diabetes
**CV events: IFG/IGT-WHO**												
Saydah, 2001	✓	✓	✓	✓	✓	✓	✓		✓	✓		
Rodriguez, 2002	✓	✓			✓		✓		✓	✓		fibrinogen
Kowall, 2011	✓	✓			✓	✓	✓	✓	✓	✓	✓	parental diabetes
Evans, 2015	✓	✓		✓								
Hu, 2003	✓	✓			✓	✓			✓	✓		center
Nakagami, 2004	✓	✓			✓	✓			✓	✓		cohort
Eastwood, 2015	✓	✓			✓	✓			✓	✓		
**CV events: IFG/HbA1c-ADA**												
Tang, 2019	✓	✓	✓	✓								family history of diabetes
Hubbard, 2019	✓	✓		✓	✓	✓	✓			✓	✓	aspirin use
Rhee, 2016	✓	✓			✓	✓	✓	✓	✓		✓	study center, year of screening examination, family history of diabetes
Kim, 2016	✓	✓			✓	✓			✓	✓		family history of CVD

	Confounders											
	**Age**	**Sex**	**Ethnicity[a]**	**Education/income**	**BMI/ waist**	**Smoking**	**Physical activity**	**Alcohol intake**	**Blood pressure/ treatment**	**Blood lipids/ treatment**	**Chronic diseases**	**Further confounders**
Schöttker, 2013	✓	✓				✓			✓	✓		
Vistisen, 2018	✓	✓	✓			✓			✓	✓	✓	
**CV events: IFG/HbA1c/IGT-ADA**												
Wang, 2019	✓	✓		✓							✓	family history of diabetes
Ares, 2019	✓	✓			✓	✓			✓	✓	✓	eGFR
**CV events (in patients with atherosclerotic cardiovascular disease): IFG-ADA**												
Kanaya, 2005	✓	✓	✓	✓	✓	✓	✓	✓		✓	✓	overall health status, angiotensin-converting enzyme inhibitor and hormone therapy
Donahue, 2011	✓				✓	✓		✓	✓	✓	✓	
Janszky, 2009	✓	✓		✓	✓		✓		✓	✓	✓	apo B∕apo A ratio, Q wave infarction and education
Silbernagel, 2011	✓	✓			✓	✓			✓	✓	✓	eGFR, Friesinger score, glycated hemoglobin
Ding, 2014	✓	✓		✓	✓	✓	✓	✓	✓	✓	✓	marriage, glomerular filtration rate, antiplatelet drugs
Slezak, 2018	✓	✓			✓	✓			✓	✓		survey
**CV events (in patients with atherosclerotic cardiovascular disease): IFG-WHO**												
Kiviniemi, 2019	✓	✓			✓				✓			grading for angina pectoris, SYNTAX Score, and LVEF
Shahim, 2017	✓	✓		✓	✓	✓	✓		✓	✓		HADS anxiety and depression score
Nigam, 2007	✓	✓			✓	✓			✓	✓	✓	
Tamita, 2012	✓								✓	✓	✓	Previous surgery, HbA1c, piuretics,
Held, 2005	✓					✓			✓	✓	✓	
**CV events (in patients with atherosclerotic cardiovascular disease): IGT-ADA/WHO**												

	Confounders											
	**Age**	**Sex**	**Ethnicity[a]**	**Education/income**	**BMI/ waist**	**Smoking**	**Physical activity**	**Alcohol intake**	**Blood pressure/ treatment**	**Blood lipids/ treatment**	**Chronic diseases**	**Further confounders**
Kiviniemi, 2019	✓	✓			✓				✓			grading for angina pectoris, SYNTAX Score, and LVEF
Shahim, 2017	✓	✓		✓	✓	✓	✓		✓	✓		HADS anxiety and depression score
George, 2015	✓	✓				✓			✓	✓	✓	discharge diagnosis
Ding, 2014	✓	✓		✓	✓	✓	✓	✓	✓	✓	✓	marriage, glomerular filtration rate, antiplatelet drugs
Von Birgelen, 2018	✓	✓								✓	✓	
Tamita, 2012	✓								✓	✓	✓	Previous surgery, HbA1c, piuretics,
**CV events (in patients with atherosclerotic cardiovascular disease): HbA1c-ADA**												
Shin, 2016	✓	✓			✓	✓			✓	✓	✓	Killip class, LVEF, peak troponin I, serum creatinine, peak creatine kinase 2 isoenzyme, treated vessel, lesion type, PCI, CRP
Shahim, 2017	✓	✓		✓	✓	✓	✓		✓	✓		HADS anxiety and depression score
Wang, 2020	✓	✓			✓				✓	✓	✓	creatinine clearance rate
Choi, 2018	✓	✓				✓			✓	✓	✓	bifurcation Lesion
**CV events (in patients with atherosclerotic cardiovascular disease): IFG/HbA1c-ADA**												
Farhan, 2019	✓	✓									✓	minimal luminal area, prior PCI
Kim, 2020	✓	✓			✓	✓			✓	✓	✓	PCI, CABG, CK-MB, NT-ProBNP, creatinine, eGFR, medicine, vessel disease, stent
**CV events (in patients with atherosclerotic cardiovascular disease): IFG/HbA1c/IGT-ADA**												
Jin, 2019	✓	✓			✓	✓			✓	✓	✓	family history of CAD, gensini score, LVEF, creatinine, NT-proBNP, big ET-1, fifibrinogen
Bjarnason, 2019	✓	✓			✓	✓			✓		✓	
**CV mortality: IFG-ADA**												

	Confounders											
	**Age**	**Sex**	**Ethnicity[a]**	**Education/income**	**BMI/ waist**	**Smoking**	**Physical activity**	**Alcohol intake**	**Blood pressure/ treatment**	**Blood lipids/ treatment**	**Chronic diseases**	**Further confounders**
	No information in the paper/meta-analysis which studies and confounders were included; no access to the supplement and authors did not reply to e-mail request											
**CV mortality: IFG-WHO**												
	No information in the paper/meta-analysis which studies and confounders were included; no access to the supplement and authors did not reply to e-mail request											
**CV mortality: IGT-ADA/WHO**												
	No information in the paper/meta-analysis which studies and confounders were included; no access to the supplement and authors did not reply to e-mail request											
**CVD incidence: IFG-ADA**												
	No information in the paper/meta-analysis which studies and confounders were included; no access to the supplement and authors did not reply to e-mail request											
**CVD incidence: IFG-WHO**												
	No information in the paper/meta-analysis which studies and confounders were included; no access to the supplement and authors did not reply to e-mail request											
**CVD incidence: IGT-ADA/WHO**												
	No information in the paper/meta-analysis which studies and confounders were included; no access to the supplement and authors did not reply to e-mail request											
**CHD: IFG-ADA**												
Doi, 2010	✓	✓			✓	✓	✓	✓	✓	✓		electrocardiogram abnormalities
Kim, 2016	✓	✓			✓	✓			✓	✓		family history of CVD
Ma, 2012	✓	✓		✓	✓			✓	✓			
Kim, 2008	✓	✓										
Deedwania, 2013	✓	✓	✓	✓	✓	✓	✓	✓	✓	✓	✓	ankle arm index ratio, haemoglobin, albumin, uric acid, serum insulin, LV hypertrophy, atrial fibrillation, bundle branch block, CRP
Yeboah, 2011	✓	✓	✓		✓	✓			✓	✓		
Kim, 2013	✓				✓	✓			✓	✓		family history of CVD

	Confounders											
	**Age**	**Sex**	**Ethnicity[a]**	**Education/income**	**BMI/ waist**	**Smoking**	**Physical activity**	**Alcohol intake**	**Blood pressure/ treatment**	**Blood lipids/ treatment**	**Chronic diseases**	**Further confounders**
Wang, 2007	✓	✓		✓		✓				✓		family history of diabetes
Samaras, 2015	✓	✓			✓	✓			✓			
Selvin, 2013	✓	✓	✓	✓	✓	✓	✓	✓	✓	✓		family history of diabetes
McNeill, 2006	✓	✓	✓									
Levitzky, 2008	✓	✓			✓	✓			✓	✓		
Khang, 2010	✓	✓			✓				✓	✓		
Tai, 2004	✓	✓	✓									
Liu, 2007	✓	✓				✓				✓		CVD family history
Kokubo, 2010	✓	✓			✓	✓		✓	✓	✓		
Mathenge, 2020	✓	✓	✓	✓	✓	✓	✓		✓	✓		aspirin use, cognitive function
Parizadeh, 2019	✓			✓	✓	✓			✓	✓	✓	family history of diabetes, eGFR
Kim, 2017	✓	✓		✓	✓	✓	✓	✓	✓	✓		antithrombotics
Ahn, 2018	✓				✓	✓			✓	✓		
Mongraw-Chaffin, 2017	✓	✓	✓	✓	✓	✓			✓	✓		diabetes treatment
Saito, 2011	✓	✓			✓	✓	✓	✓	✓	✓		community
**CHD: IFG-WHO**												
Oizumi, 2008	✓	✓							✓			
Doi, 2010	✓	✓			✓	✓	✓	✓	✓	✓		electrocardiogram abnormalities
Onat, 2013	✓	✓			✓	✓			✓	✓		CRP

	Confounders											
	**Age**	**Sex**	**Ethnicity[a]**	**Education/income**	**BMI/ waist**	**Smoking**	**Physical activity**	**Alcohol intake**	**Blood pressure/ treatment**	**Blood lipids/ treatment**	**Chronic diseases**	**Further confounders**
Palmieri, 2006	✓	✓										center
DECODE, 2001	✓	✓			✓	✓			✓	✓		center
McNeill, 2005	✓	✓			✓	✓	✓		✓		✓	self-reported health
Wannamethee, 2008	✓	✓		✓		✓	✓	✓				
Wang, 2007	✓	✓		✓		✓				✓		family history of diabetes
Levitzky, 2008	✓	✓			✓	✓			✓	✓		
McNeill, 2006	✓	✓	✓									
Parizadeh, 2019	✓			✓	✓	✓			✓	✓	✓	family history of diabetes, eGFR
Brunner, 2010	✓	✓										
**CHD: IGT-ADA/WHO**												
Pankow, 2007	✓	✓	✓		✓	✓			✓	✓		
Doi, 2010	✓	✓			✓	✓	✓	✓	✓	✓		electrocardiogram abnormalities
Wang, 2007	✓	✓		✓		✓				✓		family history of diabetes
Oizumi, 2008	✓	✓							✓			
DECODE, 2001	✓	✓			✓	✓			✓	✓		center
Kim, 2008	✓	✓										
Tai, 2004	✓	✓	✓									
Onat, 2013	✓	✓			✓	✓			✓	✓		CRP
Fang, 2019	✓	✓			✓	✓			✓	✓	✓	insulin resistance, eGFR

	Confounders											
	**Age**	**Sex**	**Ethnicity[a]**	**Education/income**	**BMI/ waist**	**Smoking**	**Physical activity**	**Alcohol intake**	**Blood pressure/ treatment**	**Blood lipids/ treatment**	**Chronic diseases**	**Further confounders**
Parizadeh, 2019	✓			✓	✓	✓			✓	✓	✓	family history of diabetes, eGFR
Smith, 2002	✓	✓	✓									
**CHD: HbA1c-ADA**												
Eastwood, 2015	✓	✓			✓	✓			✓	✓		
Kim, 2016	✓	✓			✓	✓			✓	✓		family history of CVD
Selvin, 2013	✓	✓	✓	✓	✓	✓	✓	✓	✓	✓		family history of diabetes
**CHD: IFG/IGT-WHO**												
Eastwood, 2015	✓	✓			✓	✓			✓	✓		
Wang, 2007	✓	✓		✓		✓				✓		family history of diabetes
Madssen, 2012	✓	✓			✓	✓	✓		✓		✓	
Doi, 2010	✓	✓			✓	✓	✓	✓	✓	✓		electrocardiogram abnormalities
Bonora, 2003	✓	✓		✓		✓	✓	✓		✓	✓	
**CHD: IFG/HbA1c-ADA**												
Kim, 2016	✓	✓			✓	✓			✓	✓		family history of CVD
Hubbard, 2019	✓	✓		✓	✓	✓	✓			✓	✓	aspirin use
**CHD (in patients with atherosclerotic cardiovascular disease): IFG-ADA**												
Kanaya, 2005	✓	✓	✓	✓	✓	✓	✓	✓		✓	✓	overall health status, angiotensin-converting enzyme inhibitor and hormone therapy
Janszky, 2009	✓	✓		✓	✓		✓		✓	✓	✓	apo B ⁄ apo A ratio, Q wave infarction and education
**CHD (in patients with atherosclerotic cardiovascular disease): IFG-WHO**												

	Confounders											
	Age	Sex	Ethnicity[a]	Education/income	BMI/ waist	Smoking	Physical activity	Alcohol intake	Blood pressure/ treatment	Blood lipids/ treatment	Chronic diseases	Further confounders
Kiviniemi, 2019	✓	✓			✓				✓			grading for angina pectoris, SYNTAX Score, and LVEF
Fisman, 2001	✓	✓			✓	✓				✓	✓	functional class, peripheral vascular disease, anginal syndrome
**CHD (in patients with atherosclerotic cardiovascular disease): IGT-ADA/WHO**												
Kiviniemi, 2019	✓	✓			✓				✓			grading for angina pectoris, SYNTAX Score, and LVEF
George, 2015	✓	✓				✓			✓	✓	✓	discharge diagnosis
Von Birgelen, 2018	✓	✓								✓	✓	
**CHD (in patients with atherosclerotic cardiovascular disease): HbA1c-ADA**												
Shin, 2016	✓	✓			✓	✓			✓	✓	✓	Killip class, LVEF, peak troponin I, serum creatinine, peak creatine kinase 2 isoenzyme, treated vessel, lesion type, PCI, CRP
Wang, 2020	✓	✓			✓				✓	✓	✓	creatinine clearance rate
**Stroke: IFG-ADA**												
Doi, 2010	✓	✓			✓	✓	✓	✓	✓	✓		electrocardiogram abnormalities
Yeboah, 2011	✓	✓	✓		✓	✓			✓	✓		
Deedwania, 2013	✓	✓	✓	✓	✓	✓	✓	✓	✓	✓	✓	ankle arm index ratio, haemoglobin, albumin, uric acid, serum insulin, LV hypertrophy, atrial fibrillation, bundle branch block, CRP
Khang, 2010	✓	✓			✓				✓	✓		
Kim, 2016	✓	✓			✓	✓			✓	✓		family history of CVD
Kim, 2013	✓				✓	✓			✓	✓		family history of CVD
Ma, 2012	✓	✓		✓	✓			✓	✓			
Sung, 2009	✓	✓		✓	✓	✓	✓	✓	✓	✓		height, area of residence

	Confounders											
	Age	Sex	Ethnicity[a]	Education/income	BMI/ waist	Smoking	Physical activity	Alcohol intake	Blood pressure/ treatment	Blood lipids/ treatment	Chronic diseases	Further confounders
Kokubo, 2010	✓	✓			✓	✓		✓	✓	✓		
Liu, 2007	✓	✓				✓				✓		CVD family history
Samaras, 2015	✓	✓			✓	✓			✓			
Selvin, 2013	✓	✓	✓	✓	✓	✓	✓	✓	✓	✓		family history of diabetes
Kim, 2017	✓	✓		✓	✓	✓	✓	✓	✓	✓		antithrombotics
Mongraw-Chaffin, 2017	✓	✓	✓	✓	✓	✓			✓	✓		diabetes treatment
Wang, 2008	✓	✓			✓							
Parizadeh, 2019	✓			✓	✓	✓			✓	✓	✓	family history of diabetes, eGFR
**Stroke: IFG-WHO**												
Doi, 2010	✓	✓			✓	✓	✓	✓	✓	✓		electrocardiogram abnormalities
Oizumi, 2008	✓	✓							✓			
Sung, 2009	✓	✓		✓	✓	✓	✓	✓	✓	✓		height, area of residence
Hyvärinen, 2009	✓	✓			✓	✓			✓	✓		center
Sui, 2011	✓	✓			✓	✓		✓	✓	✓		family history of CVD, abnormal ECG
Mazza, 2001	✓					✓			✓		✓	atrial fibrillation, LV hypertrophy, uric acid, serum potassium, sodium
Wang, 2008	✓	✓			✓							
Parizadeh, 2019	✓			✓	✓	✓			✓	✓	✓	family history of diabetes, eGFR
**Stroke: IGT-ADA/WHO**												
Doi, 2010	✓	✓			✓	✓	✓	✓	✓	✓		electrocardiogram abnormalities

	Confounders											
	Age	Sex	Ethnicity[a]	Education/income	BMI/ waist	Smoking	Physical activity	Alcohol intake	Blood pressure/ treatment	Blood lipids/ treatment	Chronic diseases	Further confounders
Hyvärinen, 2009	✓	✓			✓	✓			✓	✓		center
Kaarisalo, 2006		✓				✓			✓		✓	Previous stroke/TIA, perceived health status, cardiac failure, atrial fibrillation, claudification, acetylsalisylic acid
Oizumi, 2008	✓	✓							✓			
Parizadeh, 2019	✓			✓	✓	✓			✓	✓	✓	family history of diabetes, eGFR
Smith, 2002	✓	✓	✓									
Wang, 2008	✓	✓			✓							
Fang, 2019	✓	✓			✓	✓			✓	✓	✓	insulin resistance, eGFR
**Stroke: HbA1c-ADA**												
Selvin, 2013	✓	✓	✓	✓	✓	✓	✓	✓	✓	✓		family history of diabetes
Wang, 2011	✓	✓				✓			✓	✓		log urinary albumin:creatinine ratio, baseline FBG levels
Birkenhager-Gillesse, 2015		✓		✓	✓	✓		✓	✓	✓	✓	living conditions, creatinine clearance, CRP
Goto, 2015	✓	✓			✓	✓	✓	✓	✓	✓		
**Stroke: IFG/IGT-WHO**												
Eastwood, 2015	✓	✓			✓	✓			✓	✓		
Zhang, 2008	✓	✓			✓	✓	✓	✓	✓	✓		micro- and macro-albuminuria
**Stroke: IFG/HbA1c-ADA**												
Kim, 2016	✓	✓			✓	✓			✓	✓		family history of CVD
Hubbard, 2019	✓	✓		✓	✓	✓	✓			✓	✓	aspirin use
**Stroke (in patients with atherosclerotic cardiovascular disease): IFG-ADA**												

	Confounders											
	Age	Sex	Ethnicity[a]	Education/income	BMI/ waist	Smoking	Physical activity	Alcohol intake	Blood pressure/ treatment	Blood lipids/ treatment	Chronic diseases	Further confounders
Kanaya, 2005	✓	✓	✓	✓	✓	✓	✓	✓		✓	✓	overall health status, angiotensin-converting enzyme inhibitor and hormone therapy
Janszky, 2009	✓	✓		✓	✓		✓		✓	✓	✓	apo B ⁄ apo A ratio, Q wave infarction and education
**Atrial fibrillation: IFG-ADA/WHO**												
Kokubo, 2017	✓	✓										
Lee, 2017	✓	✓				✓		✓			✓	
Huxley, 2011	✓	✓	✓	✓	✓	✓			✓			height
**Atrial fibrillation: IFG-ADA**												
Kokubo, 2017	✓	✓										
Lee, 2017	✓	✓				✓		✓			✓	
Huxley, 2011	✓	✓	✓	✓	✓	✓			✓			height
**Heart failure: IFG-ADA**												
Janszky, 2009	✓	✓		✓	✓		✓		✓	✓	✓	apo B ⁄ apo A ratio, Q wave infarction and education
Wang, 2010	✓	✓				✓	✓	✓	✓	✓	✓	
Nichols, 2009	✓	✓			✓	✓			✓	✓	✓	eGFR,
Mongraw-Chaffin, 2017	✓		✓	✓	✓	✓			✓	✓		
Deedwania, 2013	✓	✓	✓	✓	✓	✓	✓	✓	✓	✓	✓	ankle arm index ratio, haemoglobin, albumin, uric acid, serum insulin, LV hypertrophy, atrial fibrillation, bundle branch block, CRP
Kanaya, 2005	✓	✓	✓	✓	✓	✓	✓	✓		✓	✓	overall health status, angiotensin-converting enzyme inhibitor and hormone therapy
Held, 2007	✓	✓			✓	✓			✓	✓	✓	creatinine
Matsushita, 2010	✓	✓	✓	✓	✓	✓		✓	✓	✓	✓	eGFR, FPG, HbA1c

	Confounders											
	Age	Sex	Ethnicity[a]	Education/income	BMI/ waist	Smoking	Physical activity	Alcohol intake	Blood pressure/ treatment	Blood lipids/ treatment	Chronic diseases	Further confounders
Voulgari, 2011	✓	✓				✓	✓		✓	✓	✓	function on echocardiography
Rhee, 2020	✓	✓		✓		✓	✓	✓	✓	✓	✓	duration of diabetes
**Heart failure: IFG-WHO**												
Kanaya, 2005	✓	✓	✓	✓	✓	✓	✓	✓		✓	✓	overall health status, angiotensin-converting enzyme inhibitor and hormone therapy
Kiviniemi, 2019	✓	✓			✓				✓			grading for angina pectoris, SYNTAX Score, and LVEF
Matsushita, 2010	✓	✓	✓	✓	✓	✓		✓	✓	✓	✓	eGFR, FPG, HbA1c
Nichols, 2009	✓	✓			✓	✓			✓	✓	✓	eGFR,
Bibbins-Domingo, 2004	✓	✓			✓	✓			✓	✓	✓	creatinine clearance, left bundle-branch block
Wang, 2010	✓					✓	✓	✓	✓	✓	✓	
**Heart failure: IGT-ADA/WHO**												
Wang, 2010	✓					✓	✓	✓	✓	✓	✓	
Kiviniemi, 2019	✓	✓			✓				✓			grading for angina pectoris, SYNTAX Score, and LVEF
Fang, 2019	✓	✓			✓	✓			✓	✓	✓	insulin resistance, eGFR,
**Sudden cardiac death: IFG/IGT-ADA**												
Khosravi, 2017	✓	✓										
Laukkanen, 2013	✓	✓				✓		✓	✓	✓	✓	
**Stroke (in pts with history of stroke/TIA): IFG/IGT-ADA/WHO**												
Vermeer, 2006	✓	✓				✓			✓			minor ischaemic stroke in history
Pan, 2016	✓	✓				✓			✓	✓		history of IS, TIA, myocardial infarction, angina, congestive heart failure, known atrial fibrillation or flutter, valvular heart

	Confounders											
	**Age**	**Sex**	**Ethnicity[a]**	**Education/income**	**BMI/ waist**	**Smoking**	**Physical activity**	**Alcohol intake**	**Blood pressure/ treatment**	**Blood lipids/ treatment**	**Chronic diseases**	**Further confounders**
												disease, NIHSS on admission, time to randomisation, antiplatelet drugs
Zhu, 2017	✓	✓				✓		✓	✓	✓		time from onset to hospitalization, ischemic stroke subtypes, baseline NIHSS score.
**Stroke (in pts with history of stroke/TIA): IFG-WHO**												
Pan, 2016	✓	✓				✓			✓	✓		history of IS, TIA, myocardial infarction, angina, congestive heart failure, known atrial fibrillation or flutter, valvular heart disease, NIHSS on admission, time to randomisation, antiplatelet drugs
Zhu, 2017	✓	✓				✓		✓	✓	✓		time from onset to hospitalisation, ischaemic stroke subtypes, baseline NIHSS score.
**Poor outcome (defined as degree of disability or dependence in the daily activities of people who have suffered a stroke or other causes of neurological disability of stroke (in pts with history of stroke/TIA): IFG/IGT/HbA1c-ADA/WHO**												
Tanaka, 2013	✓	✓			✓	✓			✓	✓		baseline NIHSS, stroke subtype, atrial fibrillation, thrombolytic therapy, admission blood glucose levels
Roquer, 2014	✓											NIHSS and previous mRS
Pan, 2016	✓	✓				✓			✓	✓		history of IS, TIA, myocardial infarction, angina, congestive heart failure, known atrial fibrillation or flutter, valvular heart disease, NIHSS on admission, time to randomization, antiplatelet drugs
Osei, 2016	✓	✓							✓			family history of T2D, NIHSS score on admission, atrial fibrillation, time from stroke onset to IAT,
Zhu, 2017	✓	✓				✓		✓	✓	✓		time from onset to hospitalization, ischemic stroke subtypes, baseline NIHSS score.
**Poor outcome (defined as degree of disability or dependence in the daily activities of people who have suffered a stroke or other causes of neurological disability of stroke (in pts with history of stroke/TIA): IFG-WHO**												
Pan, 2016	✓	✓				✓			✓	✓		history of IS, TIA, myocardial infarction, angina, congestive heart failure, known atrial fibrillation or flutter, valvular heart disease, NIHSS on admission, time to randomization, antiplatelet drugs
Zhu, 2017	✓	✓				✓		✓	✓	✓		time from onset to hospitalization, ischemic stroke subtypes, baseline NIHSS score.
**Mortality of stroke (in pts with history of stroke/TIA): IFG/ IGT-ADA/WHO**												

	Confounders											
	Age	Sex	Ethnicity[a]	Education/income	BMI/ waist	Smoking	Physical activity	Alcohol intake	Blood pressure/ treatment	Blood lipids/ treatment	Chronic diseases	Further confounders
Roquer, 2014	✓											NIHSS and previous mRS
Pan, 2016	✓	✓				✓			✓	✓		history of IS, TIA, myocardial infarction, angina, congestive heart failure, known atrial fibrillation or flutter, valvular heart disease, NIHSS on admission, time to randomization, antiplatelet drugs
Zhu, 2017	✓	✓				✓		✓	✓	✓		time from onset to hospitalisation, ischaemic stroke subtypes, baseline NIHSS score
Lorea, 2017	✓	✓										reason for admission, previous myocardial infarction, glucose, blood pressure in emergency, NIHSS
**Mortality of stroke (in pts with history of stroke/TIA): IFG-WHO**												
Pan, 2016	✓	✓				✓			✓	✓		history of IS, TIA, myocardial infarction, angina, congestive heart failure, known atrial fibrillation or flutter, valvular heart disease, NIHSS on admission, time to randomisation, antiplatelet drugs
Zhu, 2017	✓	✓				✓		✓	✓	✓		time from onset to hospitalization, ischemic stroke subtypes, baseline NIHSS score.
**Mortality of stroke (in pts with history of stroke/TIA): HbA1c–ADA**												
Roquer, 2014	✓											NIHSS and previous mRS
Lorea, 2017	✓	✓										reason for admission, previous myocardial infarction, glucose, blood pressure in emergency, NIHSS
**Major adverse cardiac events: IFG/ IGT/HbA1c-ADA/WHO**												
Porter, 2008	✓										✓	Killip Class, LVEF, renal function, anemia
Fefer, 2008	✓	✓							✓		✓	Killip Class, number of diseased vessels, LVEF
De la Hera, 2009	✓									✓		indication of PCI, three-vessel or LM-CAD, LVEF, treatment with drug-eluting stents and IIb/IIIa inhibitors
Knudsen, 2011	✓	✓			✓	✓			✓	✓	✓	Troponin T and infarct size expressed as percent of ventricular mass
Kuramitsu, 2013	✓	✓								✓	✓	

	Confounders											
	Age	Sex	Ethnicity[a]	Education/income	BMI/ waist	Smoking	Physical activity	Alcohol intake	Blood pressure/ treatment	Blood lipids/ treatment	Chronic diseases	Further confounders
Shin, 2016	✓	✓			✓					✓		LVEF, Killip class, troponin I
Samir, 2016	✓	✓			✓	✓			✓	✓		LVEF, Killip class, troponin I, coronary lesion features
Von Birgelen, 2018	✓	✓								✓	✓	previous revascularization
Choi, 2018	✓	✓			✓	✓			✓	✓	✓	multivessel CAD and LVEF
Farhan, 2019	✓	✓										presence of thin-cap fibroatheroma, presence of minimal luminal area <4 mm² and prior PCI
**Major adverse cardiac events: IGT-ADA/WHO**												
Kuramitsu, 2013	✓	✓								✓	✓	
Von Birgelen, 2018	✓	✓								✓	✓	previous revascularization
**Major adverse cardiac events: HbA1c-ADA**												
Shin, 2016	✓	✓			✓					✓		LVEF, Killip class, troponin I
Samir, 2016	✓	✓			✓	✓			✓	✓		LVEF, Killip class, troponin I, coronary lesion features
Choi, 2018	✓	✓			✓	✓			✓	✓	✓	multivessel CAD and LVEF
**Chronic kidney disease: IFG-ADA/WHO**												
Fox, 2005	✓	✓			✓	✓			✓	✓	✓	baseline GFR
Kurella, 2005	✓	✓	✓									
Lucove, 2008	✓	✓		.		✓						
Rashidi, 2007	Not reported											
Sun, 2010	✓	✓		.		✓						center
Ryu, 2009	✓	✓			✓				✓	✓		baseline eGFR, GGT, uric acid

	Confounders											
	**Age**	**Sex**	**Ethnicity[a]**	**Education/income**	**BMI/ waist**	**Smoking**	**Physical activity**	**Alcohol intake**	**Blood pressure/ treatment**	**Blood lipids/ treatment**	**Chronic diseases**	**Further confounders**
Wanatable, 2012	✓	✓										
Schöttker, 2013	✓	✓			✓	✓			✓	✓	✓	eGFR
**Chronic kidney disease: IFG-WHO**												
Kurella, 2005	✓	✓	✓									
Lucove, 2008	✓	✓		.		✓						
Rashidi, 2007	Not reported											
Ryu, 2009	✓	✓			✓				✓	✓		baseline eGFR, GGT, uric acid
Wanatabe, 2012	✓	✓							✓		✓	
Schöttker, 2013	✓	✓			✓	✓			✓	✓	✓	eGFR
**Chronic kidney disease: IFG-ADA**												
Schöttker, 2013	✓	✓			✓	✓			✓	✓	✓	eGFR
Sun, 2010	✓	✓				✓						center
Selvin, 2011	✓	✓	✓	✓	✓	✓	✓	✓	✓	✓		family history of diabetes, any ETDRS for retinopathy
Xing, 2014	✓	✓	✓		✓	✓			✓	✓		
**Chronic kidney disease: HbA1c-ADA**												
Xing, 2014	✓	✓	✓		✓	✓			✓	✓		
Selvin, 2011	✓	✓	✓	✓	✓	✓	✓	✓	✓	✓		family history of diabetes, any ETDRS for retinopathy
Schöttker, 2013	✓	✓			✓	✓			✓	✓	✓	eGFR
**Total cancer: IFG-ADA/WHO**												

	Confounders											
	Age	Sex	Ethnicity[a]	Education/income	BMI/ waist	Smoking	Physical activity	Alcohol intake	Blood pressure/ treatment	Blood lipids/ treatment	Chronic diseases	Further confounders
Hirakawa, 2012	✓	✓			✓	✓	✓	✓		✓		family history of cancer, dietary factors
Balkau, 2002	✓	✓			✓				✓	✓		
Harding, 2012	✓	✓		✓	✓	✓		✓	✓			
Lu, 2003	✓	✓			✓	✓	✓	✓	✓	✓		insulin
Stattin, 2007	✓					✓						year of recruitment, fasting time
**Total cancer: IFG-WHO**												
Balkau, 2002	✓	✓			✓				✓	✓		
Harding, 2012	✓	✓		✓	✓	✓		✓	✓			
Lu, 2003	✓	✓			✓	✓	✓	✓	✓	✓		insulin
Stattin, 2007	✓					✓						year of recruitment, fasting time
**Total cancer: IGT-ADA/WHO**												
Harding, 2012	✓	✓		✓	✓	✓		✓				
Hirakawa, 2012	✓	✓			✓	✓	✓	✓		✓		family history of cancer, dietary factors
Stengard, 1992	✓	✓			✓	✓			✓	✓		functional capacity
Saydah, 2003	✓	✓	✓	✓	✓	✓	✓	✓	✓	✓		
Stattin, 2007	✓					✓						year of recruitment, fasting time
Perseghin, 2012	✓	✓										
**Total cancer: IFG/IGT-WHO**												
Zhou, 2010	✓	✓			✓	✓			✓	✓		

	Confounders											
	**Age**	**Sex**	**Ethnicity[a]**	**Education/income**	**BMI/ waist**	**Smoking**	**Physical activity**	**Alcohol intake**	**Blood pressure/ treatment**	**Blood lipids/ treatment**	**Chronic diseases**	**Further confounders**
Kowall, 2011	✓	✓			✓	✓	✓	✓	✓	✓	✓	family history of T2D
**Stomach/colorectal cancer: IFG/IGT-ADA/WHO**												
Hirakawa, 2012	✓	✓			✓	✓	✓	✓		✓		family history of cancer, dietary factors
Parekh, 2013	✓	✓			✓	✓		✓				
Zhou, 2010	✓	✓			✓	✓			✓	✓		
**Liver cancer: IFG/IGT-ADA/WHO**												
Gwack, 2007	✓	✓			✓	✓		✓				hepatitis B surface antigen seropositivity
Hirakawa, 2012	✓	✓			✓	✓	✓	✓		✓		family history of cancer, dietary factors
Zhou, 2010	✓	✓			✓	✓			✓	✓		
**Hepatocellular carcinoma: IFG/IGT-ADA/WHO**												
Zhou, 2010	✓	✓			✓	✓			✓	✓		normal glucose tolerance
Batty, 2004	✓	✓			✓	✓	✓		✓	✓	✓	marital status, unexplained weight loss, triceps skinfold thickness, height adjusted FEV1
Hirakawa, 2012	✓	✓										
Chao, 2011	✓	✓				✓		✓				
Jee, 2005	✓	✓				✓		✓				first-degree family history of HCC, and baseline viral factors
**Hepatocellular carcinoma: IFG-ADA**												
Hirakawa, 2012	✓	✓										
Chao, 2011	✓	✓				✓		✓				

	Confounders											
	**Age**	**Sex**	**Ethnicity[a]**	**Education/income**	**BMI/ waist**	**Smoking**	**Physical activity**	**Alcohol intake**	**Blood pressure/ treatment**	**Blood lipids/ treatment**	**Chronic diseases**	**Further confounders**
Jee, 2005	✓	✓				✓		✓				first-degree family history of HCC, and baseline viral factors
**Bronchus and lung cancer: IFG/IGT-ADA/WHO**												
Hirakawa, 2012	✓	✓			✓	✓	✓	✓		✓		family history of cancer, dietary factors
Zhou, 2010	✓	✓			✓	✓			✓	✓		
**Prostate cancer: IFG/IGT-ADA/WHO**												
Chung, 2009	✓											
Parekh, 2013	✓	✓			✓	✓		✓				
Zhou, 2010	✓	✓			✓	✓			✓	✓		
**Kidney and bladder cancer: IFG/IGT-ADA/WHO**												
Chung, 2009	✓											
Zhou, 2010	✓	✓			✓	✓			✓	✓		
**Breast cancer: IFG/IGT-ADA/WHO**												
Lambe, 2011	✓											
Mink, 2002	✓	✓	✓		✓	✓		✓				age at menarche/menopause/first live birth, family history of breast cancer, number of sisters
Parekh, 2013	✓	✓			✓	✓		✓				
Zhou, 2010	✓	✓			✓	✓			✓	✓		
**Breast cancer: IFG-WHO**												
Lambe, 2011	✓											
Parekh, 2013	✓	✓			✓	✓		✓				

	Confounders											
	Age	Sex	Ethnicity[a]	Education/income	BMI/ waist	Smoking	Physical activity	Alcohol intake	Blood pressure/ treatment	Blood lipids/ treatment	Chronic diseases	Further confounders
**Pancreatic cancer: IFG/IGT-ADA/WHO**												
Batty, 2004	✓			✓	✓		✓		✓	✓	✓	marital status, unexplained weight loss, triceps skin fold thickness, height adjusted forced expiratory volume in one second,
Jee, 2005	✓	✓				✓		✓				
Yun, 2006	✓	✓			✓		✓	✓				
Zhou, 2010	✓	✓			✓	✓			✓	✓		
Hirakawa, 2012	✓	✓			✓	✓	✓	✓		✓		family history of cancer, dietary factors
**Pancreatic cancer: IFG-ADA**												
Jee, 2005	✓	✓				✓		✓				
Yun, 2006	✓	✓			✓		✓	✓				
Hirakawa, 2012	✓	✓			✓	✓	✓	✓		✓		family history of cancer, dietary factors
**Depressive symptoms: IFG/IGT/HbA1c-ADA/WHO**												
Golden, 2008	✓	✓	✓	✓	✓							examination site
Pieper, 2011	✓	✓		✓	✓							distribution of primary care physicians
Demakakos, 2014	✓	✓		✓								baseline depressive symptoms, marital status
Okumiya, 2015	✓	✓										depressive tendency at baseline, dependence in activities of daily living
**Depressive symptoms: IFG-ADA**												
Golden, 2008	✓	✓	✓	✓	✓							examination site
Pieper, 2011	✓	✓		✓	✓							distribution of primary care physicians

	Confounders											
	Age	Sex	Ethnicity[a]	Education/income	BMI/waist	Smoking	Physical activity	Alcohol intake	Blood pressure/treatment	Blood lipids/treatment	Chronic diseases	Further confounders
**All-cause dementia: IFG/IGT-ADA/WHO**												
	No information in the paper/meta-analysis which studies and confounders were included; no access to the supplement and authors did not reply to e-mail request											
**All-cause dementia: IFG**												
	No information in the paper/meta-analysis which studies and confounders were included; no access to the supplement and authors did not reply to e-mail request											
**All-cause dementia: IGT**												
	No information in the paper/meta-analysis which studies and confounders were included; no access to the supplement and authors did not reply to e-mail request											
**Alzheimer's dementia: IFG/IGT**												
	No information in the paper/meta-analysis which studies and confounders were included; no access to the supplement and authors did not reply to e-mail request											
**Vascular dementia: IFG/IGT**												
	No information in the paper/meta-analysis which studies and confounders were included; no access to the supplement and authors did not reply to e-mail request											
**Cognitive impairment: IFG/IGT**												
	No information in the paper/meta-analysis which studies and confounders were included; no access to the supplement and authors did not reply to e-mail request											

[a] Only studies of participants with multiple ethnicities need to be adjusted for ethnicity.

ESM Table 6: Detailed description of the risk of bias assessment in the identified meta-analyses using the risk of bias in systematic reviews (ROBIS) tool

Review	Domain					Overall judgement
	Study eligibility criteria	Identification and selection of studies	Data collection and study appraisal	Synthesis and findings	Risk of bias in the review	Explanation
Aune 2018[119]						Unclear definition of prediabetes, unclear study quality, no subgroup/sensitivity analyses possible
Aune 2018[118]						Unclear definition of prediabetes, unclear if data extraction and NOS assessment were conducted by two researchers, unclear study quality, no subgroup/sensitivity analyses possible
Cai 2020[114]						Only concerns: search restricted to human studies and inclusion of duplicate cohorts, however, when we excluded the duplicate cohorts, the results did not change
Cai 2021[127]						Only concerns: search restricted to human studies
Echouffo-Tcheugui 2016[121]						
Fu 2016[125]						No adherence to reporting guidelines or checklists (e.g. MOOSE or PRISMA), unclear definition of prediabetes, no clear exclusion criteria provided, study quality not assessed
Huang 2014[123]						Search restricted to human studies, unclear if data extraction and NOS assessment were conducted by two researchers, cancer incidence and mortality pooled, no discussion of study quality

ESM Table 6: Detailed description of the risk of bias assessment in the identified meta-analyses using the risk of bias in systematic reviews (ROBIS) tool

Review	Domain					Overall judgement
	Study eligibility criteria	Identification and selection of studies	Data collection and study appraisal	Synthesis and findings	Risk of bias in the review	Explanation
Huang 2016[116]						Only concern: search restricted to human studies, otherwise low risk of bias
Li 2020[115]						Unclear if the steps of the review were conducted by two investigators independently
Mitsios 2018[117]						Search restricted to English and human studies, potentially relevant studies excluded due to missing data (no authors contacted), unclear definition of stroke
Mutie 2020[122]						No protocol, literature search only conducted on one database and restricted to human studies, unclear if two independent investigators conducted the data extraction and NOS assessment
Pan 2019[112]						Search restricted to human studies, unclear if NOS assessment was conducted by two investigators, only studies with NOS ≥7 included in data synthesis, no discussion of limitations of cohort studies
Tong 2016[126]						Unclear if data extraction and NOS assessment were conducted by two researchers, potentially relevant studies excluded due to missing data (authors not contacted), no subgroup analyses regarding different prediabetes definitions, no discussion of study quality
Xu 2017[124]						No protocol, unclear definition of prediabetes, nearly no information on literature search, unclear if NOS assessment were conducted by two researchers, use of fixed effect model, mixed prediabetes definitions, mixed study designs (prospective and retrospective studies)

ESM Table 6: Detailed description of the risk of bias assessment in the identified meta-analyses using the risk of bias in systematic reviews (ROBIS) tool

Review	Domain					Overall judgement
	**Study eligibility criteria**	**Identification and selection of studies**	**Data collection and study appraisal**	**Synthesis and findings**	**Risk of bias in the review**	**Explanation**
Xue 2019[113]						No adherence to reporting guidelines or checklists (e.g. MOOSE or PRISMA), unclear definition of prediabetes and dementia, search restricted to English studies, only one database searched, some studies might be missing
Zhao 2020[120]						No protocol, exclusion criteria not defined, 4 studies excluded due to missing data and authors not contacted to receive missing information, literature search restricted to human and English studies

low risk; high risk; unclear risk;

NOS, Newcastle-Ottawa scale.

ESM Table 7: Certainty of evidence of the included meta-analyses by using the GRADE tool

Certainty assessment							Effect	Certainty
No of studies	Study design	Risk of bias	Inconsistency	Indirectness	Imprecision	Other considerations	Summary HR (95% confidence interval)	
**All-cause mortality**								
**All-cause mortality: IFG-ADA**								
18	observational studies	not serious	not serious	not serious	not serious	dose response gradient [a,b,c]	**HR 1.08** (1.03 to 1.13)	⊕⊕⊕◯ MODERATE
**All-cause mortality: IFG-WHO**								
19	observational studies	not serious	not serious	not serious	not serious	dose response gradient [a,b,c]	**HR 1.13** (1.05 to 1.20)	⊕⊕⊕◯ MODERATE
**All-cause mortality: IGT-ADA/WHO**								
15	observational studies	not serious	not serious	not serious	not serious	dose response gradient [a,b,c]	**HR 1.25** (1.17 to 1.32)	⊕⊕⊕◯ MODERATE
**All-cause mortality: HbA1c-ADA**								
7	observational studies	not serious	not serious	not serious	not serious	none	**HR 0.98** (0.91 to 1.05)	⊕⊕◯◯ LOW
**All-cause mortality: IFG/IGT-ADA**								
2	observational studies	not serious	not serious	not serious	serious [d]	dose response gradient [a,b,c]	**HR 1.14** (0.82 to 1.58)	⊕⊕◯◯ LOW
**All-cause mortality: IFG/IGT-WHO**								
8	observational studies	not serious	not serious	not serious	not serious	dose response gradient [a,b,c]	**HR 1.17** (1.13 to 1.20)	⊕⊕⊕◯ MODERATE

Certainty assessment							Effect	Certainty
No of studies	Study design	Risk of bias	Inconsistency	Indirectness	Imprecision	Other considerations	Summary HR (95% confidence interval)	
**All-cause mortality: IFG/HbA1c-ADA**								
4	observational studies	not serious	not serious	not serious	not serious	none	**HR 1.05** (0.94 to 1.16)	⨁⨁◯◯ LOW
**Long-term all-cause mortality (in patients after PCI): HbA1c-ADA**								
3	observational studies	not serious	not serious	not serious	serious [d]	none	**HR 1.18** (0.92 to 1.50)	⨁◯◯◯ VERY LOW
**Short-term all-cause mortality (in patients after PCI): HbA1c-ADA**								
2	observational studies	not serious	not serious	not serious	serious [d]	none	**HR 1.00** (0.69 to 1.44)	⨁◯◯◯ VERY LOW
**All-cause mortality in patients with atherosclerotic cardiovascular disease: IFG-ADA**								
5	observational studies	not serious	not serious	not serious	not serious	none	**HR 1.60** (1.15 to 2.22)	⨁⨁◯◯ LOW
**All-cause mortality in patients with atherosclerotic cardiovascular disease: IFG-WHO**								
5	observational studies	not serious	not serious	not serious	serious [d]	none	**HR 1.19** (0.98 to 1.45)	⨁◯◯◯ VERY LOW
**All-cause mortality in patients with atherosclerotic cardiovascular disease: IGT:ADA/WHO**								
3	observational studies	not serious	not serious	not serious	serious [d]	none	**HR 1.34** (0.94 to 1.93)	⨁◯◯◯ VERY LOW
**All-cause mortality in patients with atherosclerotic cardiovascular disease: HbA1c-ADA**								
2	observational studies	not serious	not serious	not serious	serious [d]	none	**HR 2.30** (0.56 to 9.41)	⨁◯◯◯ VERY LOW

Certainty assessment							Effect	Certainty
No of studies	Study design	Risk of bias	Inconsistency	Indirectness	Imprecision	Other considerations	Summary HR (95% confidence interval)	
**All-cause mortality in patients with atherosclerotic cardiovascular disease: IFG/IGT-ADA**								
2	observational studies	not serious	not serious	not serious	serious [d]	none	**HR 1.62** (0.96 to 2.73)	⊕◯◯◯ VERY LOW
**All-cause mortality in patients with atherosclerotic cardiovascular disease: IFG/IGT-WHO**								
2	observational studies	not serious	not serious	not serious	serious [d]	none	**HR 1.07** (0.75 to 1.52)	⊕◯◯◯ VERY LOW
**Cardiovascular outcomes & cardiovascular mortality**								
**CV events: IFG-ADA**								
22	observational studies	not serious	not serious	not serious	not serious	dose response gradient [a,b,c]	**HR 1.09** (1.03 to 1.15)	⊕⊕⊕◯ MODERATE
**CV events: IFG-WHO**								
25	observational studies	not serious	not serious	not serious	not serious	dose response gradient [a,b,c]	**HR 1.20** (1.09 to 1.34)	⊕⊕⊕◯ MODERATE
**CV events: IGT-ADA/WHO**								
19	observational studies	not serious	not serious	not serious	not serious	dose response gradient [a,b,c]	**HR 1.23** (1.13 to 1.34)	⊕⊕⊕◯ MODERATE
**CV events: HbA1c-ADA**								
8	observational studies	not serious	not serious	not serious	not serious	none	**HR 1.05** (0.97 to 1.13)	⊕⊕◯◯ LOW

Certainty assessment							Effect	Certainty
No of studies	Study design	Risk of bias	Inconsistency	Indirectness	Imprecision	Other considerations	Summary HR (95% confidence interval)	
**CV events: IFG/IGT-ADA**								
2	observational studies	not serious	not serious	not serious	serious [d]	dose response gradient [a,b,c]	**HR 1.15** (0.91 to 1.45)	⨁⨁◯◯ LOW
**CV events: IFG/IGT-WHO**								
7	observational studies	not serious	not serious	not serious	not serious	dose response gradient [a,b,c]	**HR 1.10** (0.99 to 1.21)	⨁⨁⨁◯ MODERATE
**CV events: IFG/HbA1c-ADA**								
6	observational studies	not serious	not serious	not serious	not serious	none	**HR 1.05** (0.97 to 1.13)	⨁⨁◯◯ LOW
**CV events: IFG/IGT/HbA1c-ADA**								
2	observational studies	not serious	not serious	not serious	not serious	none	**HR 0.98** (0.92 to 1.05)	⨁⨁◯◯ LOW
**CV events in patients with atherosclerotic cardiovascular disease: IFG-ADA**								
6	observational studies	not serious	not serious	not serious	not serious	dose response gradient [a,b,c]	**HR 1.33** (1.02 to 1.75)	⨁⨁⨁◯ MODERATE
**CV events in patients with atherosclerotic cardiovascular disease: IFG-WHO**								
5	observational studies	not serious	not serious	not serious	serious [d]	dose response gradient [a,b,c]	**HR 1.49** (0.99 to 2.24)	⨁⨁◯◯ LOW
**CV events in patients with atherosclerotic cardiovascular disease: IGT-ADA/WHO**								
6	observational studies	not serious	not serious	not serious	not serious	dose response gradient [a,b,c]	**HR 1.52** (1.24 to 1.85)	⨁⨁⨁◯ MODERATE

Certainty assessment							Effect	Certainty
No of studies	Study design	Risk of bias	Inconsistency	Indirectness	Imprecision	Other considerations	Summary HR (95% confidence interval)	
**CV events in patients with atherosclerotic cardiovascular disease: HbA1c-ADA**								
4	observational studies	not serious	not serious	not serious	not serious	none	**HR 1.24** (1.05 to 1.48)	⊕⊕◯◯ LOW
**CV events in patients with atherosclerotic cardiovascular disease: IFG and/or HbA1c:-ADA**								
2	observational studies	not serious	not serious	not serious	not serious	none	**HR 1.61** (1.07 to 2.43)	⊕⊕◯◯ LOW
**CV events in patients with atherosclerotic cardiovascular disease: IFG and/or HbA1c and/or IGT-ADA**								
2	observational studies	not serious	not serious	not serious	serious [d]	none	**HR 1.16** (0.86 to 1.57)	⊕◯◯◯ VERY LOW
**CVD mortality: IFG-ADA**								
6	observational studies	not serious	not serious	not serious	not serious	dose response gradient [a]	**HR 1.27** (1.02 to 1.58)	⊕⊕⊕◯ MODERATE
**CVD mortality: IFG-WHO**								
13	observational studies	not serious	not serious	not serious	not serious	dose response gradient [a]	**HR 1.20** (1.05 to 1.38)	⊕⊕⊕◯ MODERATE
**CVD mortality: IGT-ADA/WHO**								
9	observational studies	not serious	not serious	not serious	not serious	dose response gradient [a]	**HR 1.30** (1.18 to 1.44)	⊕⊕⊕◯ MODERATE
**CVD incidence: IFG-ADA**								
9	observational studies	not serious	not serious	not serious	not serious	dose response gradient [a,b]	**HR 1.10** (1.03 to 1.18)	⊕⊕⊕◯ MODERATE

Certainty assessment							Effect	Certainty
No of studies	Study design	Risk of bias	Inconsistency	Indirectness	Imprecision	Other considerations	Summary HR (95% confidence interval)	
**CVD incidence: IFG-WHO**								
5	observational studies	not serious	not serious	not serious	not serious	dose response gradient [b]	**HR 1.39** (1.15 to 1.68)	⊕⊕⊕◯ MODERATE
**CVD incidence: IGT-ADA/WHO**								
4	observational studies	not serious	not serious	not serious	not serious	dose response gradient [a,b]	**HR 1.29** (1.11 to 1.50)	⊕⊕⊕◯ MODERATE
**CHD: IFG-ADA**								
22	observational studies	not serious	not serious	not serious	not serious	dose response gradient [a,b,C]	**HR 1.09** (1.05 to 1.13)	⊕⊕⊕◯ MODERATE
**CHD: IFG-WHO**								
12	observational studies	not serious	not serious	not serious	not serious	dose response gradient [a,b,C]	**HR 1.17** (1.09 to 1.26)	⊕⊕⊕◯ MODERATE
**CHD: IGT-ADA/WHO**								
11	observational studies	not serious	not serious	not serious	not serious	dose response gradient [a,b,C]	**HR 1.21** (1.09 to 1.34)	⊕⊕⊕◯ MODERATE
**CHD: HbA1c-ADA**								
3	observational studies	not serious	not serious	not serious	not serious	none	**HR 1.30** (1.04 to 1.62)	⊕⊕◯◯ LOW
**CHD: IFG/IGT-WHO**								
5	observational studies	not serious	not serious	not serious	not serious	dose response gradient [a,b,C]	**HR 1.17** (1.02 to 1.35)	⊕⊕⊕◯ MODERATE

Certainty assessment							Effect	Certainty
No of studies	Study design	Risk of bias	Inconsistency	Indirectness	Imprecision	Other considerations	Summary HR (95% confidence interval)	
**CHD: IFG/HbA1c-ADA**								
2	observational studies	not serious	not serious	not serious	serious [d]	none	**HR 1.11** (0.88 to 1.39)	⨁⨁◯◯ LOW
**CHD in patients with atherosclerotic cardiovascular disease: IFG-ADA**								
2	observational studies	not serious	not serious	not serious	serious [d]	none	**HR 1.10** (0.92 to 1.30)	⨁◯◯◯ VERY LOW
**CHD in patients with atherosclerotic cardiovascular disease: IFG-WHO**								
2	observational studies	not serious	not serious	not serious	serious [d]	none	**HR 1.24** (0.99 to 1.56)	⨁◯◯◯ VERY LOW
**CHD in patients with atherosclerotic cardiovascular disease: IGT-ADA/WHO**								
3	observational studies	not serious	not serious	not serious	serious [d]	none	**HR 1.14** (0.84 to 1.54)	⨁◯◯◯ VERY LOW
**CHD in patients with atherosclerotic cardiovascular disease: HbA1c-ADA**								
2	observational studies	not serious	not serious	not serious	serious [d]	none	**HR 1.16** (0.65 to 2.05)	⨁◯◯◯ VERY LOW
**Stroke: IFG-ADA**								
16	observational studies	not serious	not serious	not serious	not serious	dose response gradient [a,b,C]	**HR 1.06** (1.01 to 1.11)	⨁⨁⨁◯ MODERATE
**Stroke: IFG-WHO**								
8	observational studies	not serious	not serious	not serious	not serious	dose response gradient [a,b,C]	**HR 1.18** (1.10 to 1.26)	⨁⨁⨁◯ MODERATE

Certainty assessment							Effect	Certainty
No of studies	Study design	Risk of bias	Inconsistency	Indirectness	Imprecision	Other considerations	Summary HR (95% confidence interval)	
**Stroke: IGT-ADA/WHO**								
8	observational studies	not serious	not serious	not serious	not serious	dose response gradient [a,b,c]	**HR 1.30** (1.10 to 1.54)	⊕⊕⊕◯ MODERATE
**Stroke: HbA1c**								
4	observational studies	not serious	not serious	not serious	serious [d]	dose response gradient [e]	**HR 1.19** (0.87 to 1.63)	⊕⊕◯◯ LOW
**Stroke: IFG/IGT-WHO**								
2	observational studies	not serious	not serious	not serious	serious [d]	dose response gradient [a,b,c]	**HR 1.16** (0.81 to 1.65)	⊕⊕◯◯ LOW
**Stroke: IFG and/or HbA1c-ADA**								
2	observational studies	not serious	not serious	not serious	serious [d]	none	**HR 1.01** (0.79 to 1.30)	⊕◯◯◯ VERY LOW
**Stroke in patients with atherosclerotic cardiovascular disease: IFG-ADA**								
2	observational studies	not serious	not serious	not serious	serious [d]	none	**HR 0.99** (0.63 to 1.54)	⊕◯◯◯ VERY LOW
**Atrial fibrillation: IFG-ADA or IFG-WHO**								
3	observational studies	not serious	not serious	not serious	not serious	dose response gradient [f]	**HR 1.13** (1.003 to 1.27)	⊕⊕⊕◯ MODERATE
**Atrial fibrillation: IFG-ADA**								
3	observational studies	not serious	not serious	not serious	not serious	dose response gradient [f]	**HR 1.13** (1.03 to 1.24)	⊕⊕⊕◯ MODERATE

Certainty assessment							Effect	Certainty
No of studies	Study design	Risk of bias	Inconsistency	Indirectness	Imprecision	Other considerations	Summary HR (95% confidence interval)	
**Heart failure: IFG-ADA**								
10	observational studies	not serious	not serious	not serious	not serious	dose response gradient [a,b]	**HR 1.10** (1.06 to 1.14)	⊕⊕⊕◯ MODERATE
**Heart failure: IFG-WHO**								
6	observational studies	not serious	not serious	not serious	not serious	dose response gradient [a,b]	**HR 1.18** (1.07 to 1.30)	⊕⊕⊕◯ MODERATE
**Heart failure: IGT-ADA/WHO**								
3	observational studies	not serious	not serious	not serious	not serious	dose response gradient [a,b]	**HR 1.58** (1.04 to 2.39)	⊕⊕⊕◯ MODERATE
**Sudden cardiac death: IFG/IGT-ADA**								
2	observational studies	not serious	not serious	not serious	not serious	none	**HR 1.52** (1.08 to 2.14)	⊕⊕◯◯ LOW
**Stroke in patients with history of stroke/TIA: IFG/IGT-ADA/WHO**								
3	observational studies	not serious	not serious	not serious	serious [d]	none	**HR 1.45** (0.98 to 2.14)	⊕◯◯◯ VERY LOW
**Stroke in patients with history of stroke/TIA: IFG-WHO**								
2	observational studies	not serious	not serious	not serious	serious [d]	none	**HR 1.17** (0.55 to 2.48)	⊕◯◯◯ VERY LOW

Certainty assessment							Effect	Certainty
No of studies	Study design	Risk of bias	Inconsistency	Indirectness	Imprecision	Other considerations	Summary HR (95% confidence interval)	
**Poor outcomes in patients with history of stroke/TIA: IFG/IGT/HbA1c-ADA/WHO (poor outcomes defined as degree of disability or dependence in the daily activities of people who have suffered a stroke or other causes of neurological disability)**								
5	observational studies	not serious	not serious	not serious	not serious	none	**HR 1.41** (1.01 to 1.97)	⨁⨁◯◯ LOW
**Poor outcomes in patients with history of stroke/TIA: IFG-WHO (poor outcomes defined as degree of disability or dependence in the daily activities of people who have suffered a stroke or other causes of neurological disability)**								
2	observational studies	not serious	not serious	not serious	not serious	none	**HR 1.41** (1.05 to 1.90)	⨁⨁◯◯ LOW
**Stroke mortality in patients with history of stroke/TIA: IFG/IGT-ADA/WHO**								
4	observational studies	not serious	not serious	not serious	serious [d]	none	**HR 1.40** (0.68 to 2.91)	⨁◯◯◯ VERY LOW
**Stroke mortality in patients with history of stroke/TIA: IFG-WHO**								
2	observational studies	not serious	not serious	not serious	serious [d]	none	**HR 1.64** (0.67 to 4.01)	⨁◯◯◯ VERY LOW
**Stroke mortality in patients with history of stroke/TIA: HbA1c-ADA**								
2	observational studies	not serious	serious [b]	not serious	serious [d]	none	**HR 1.28** (0.30 to 5.51)	⨁◯◯◯ VERY LOW
**MACE (in patients after PCI): IFG/IGT/HbA1c-ADA/WHO**								
10	observational studies	not serious	not serious	not serious	not serious	none	**HR 1.41** (1.14 to 1.75)	⨁⨁◯◯ LOW

Certainty assessment							Effect	Certainty
No of studies	Study design	Risk of bias	Inconsistency	Indirectness	Imprecision	Other considerations	Summary HR (95% confidence interval)	
**MACE (in patients after PCI): IGT-ADA/WHO**								
2	observational studies	not serious	not serious	not serious	not serious	none	**HR 1.62** (1.07 to 2.46)	⊕⊕◯◯ LOW
**MACE (in patients after PCI): HbA1c-ADA**								
3	observational studies	not serious	not serious	not serious	serious [d]	none	**HR 1.38** (0.88 to 2.17)	⊕◯◯◯ VERY LOW
**Microvascular outcomes**								
**Chronic kidney disease: IFG-ADA/WHO**								
8	observational studies	not serious	not serious	not serious	not serious	dose response gradient [b,g]	**HR 1.10** (1.01 to 1.20)	⊕⊕⊕◯ MODERATE
**Chronic kidney disease: IFG-WHO**								
6	observational studies	not serious	not serious	not serious	not serious	dose response gradient [b,g]	**HR 1.25** (1.02 to 1.53)	⊕⊕⊕◯ MODERATE
**Chronic kidney disease: IFG-ADA**								
4	observational studies	not serious	not serious	not serious	not serious	dose response gradient [b,g]	**HR 0.97** (0.94 to 1.01	⊕⊕◯◯ LOW
**Chronic kidney disease: HbA1c-ADA**								
3	observational studies	not serious	not serious	not serious	not serious	none	**HR 1.07** (0.94 to 1.21)	⊕⊕◯◯ LOW

Certainty assessment							Effect	Certainty
No of studies	Study design	Risk of bias	Inconsistency	Indirectness	Imprecision	Other considerations	Summary HR (95% confidence interval)	
**Cancer**								
**Total cancer: IFG-ADA or IFG-WHO**								
5	observational studies	not serious	not serious	not serious	not serious	dose response gradient [a]	**HR 1.13** (1.00 to 1.28)	⊕⊕⊕◯ MODERATE
**Total cancer: IFG-WHO**								
4	observational studies	not serious	not serious	not serious	not serious	dose response gradient [a]	**HR 1.11** (1.01 to 1.22)	⊕⊕⊕◯ MODERATE
**Total cancer: IGT-ADA/WHO**								
6	observational studies	not serious	not serious	not serious	not serious	dose response gradient [a]	**HR 1.25** (1.02 to 1.53)	⊕⊕⊕◯ MODERATE
**Total cancer: IFG/IGT-WHO**								
2	observational studies	not serious	not serious	not serious	not serious	dose response gradient [a]	**HR 1.11** (1.02 to 1.21)	⊕⊕⊕◯ MODERATE
**Stomach/colorectal cancer: IFG/IGT-ADA/WHO**								
3	observational studies	not serious	not serious	serious [h]	not serious	none	**HR 1.55** (1.15 to 2.09)	⊕◯◯◯ VERY LOW
**Liver cancer: IFG/IGT-ADA/WHO**								
3	observational studies	not serious	not serious	not serious	not serious	strong association [i]	**HR 2.01** (1.45 to 2.79)	⊕⊕⊕◯ MODERATE

Certainty assessment							Effect	Certainty
No of studies	Study design	Risk of bias	Inconsistency	Indirectness	Imprecision	Other considerations	Summary HR (95% confidence interval)	
**Hepatocellular carcinoma: IFG/IGT-ADA/WHO**								
5	observational studies	not serious	not serious	not serious	not serious	dose response gradient [j]	**HR 1.44** (1.09 to 1.90)	⊕⊕⊕◯ MODERATE
**Hepatocellular carcinoma: IFG-ADA**								
3	observational studies	not serious	not serious	not serious	not serious	dose response gradient [j]	**HR 1.23** (1.03 to 1.47)	⊕⊕⊕◯ MODERATE
**Bronchus and lung cancer: IFG/IGT-ADA/WHO**								
2	observational studies	not serious	not serious	serious [h]	serious [d]	none	**HR 1.35** (0.86 to 2.11)	⊕◯◯◯ VERY LOW
**Prostate cancer: IFG/IGT-ADA/WHO**								
3	observational studies	not serious	not serious	not serious	serious [d]	none	**HR 1.19** (0.86 to 1.65)	⊕◯◯◯ VERY LOW
**Kidney and bladder cancer: IFG/IGT-ADA/WHO**								
2	observational studies	not serious	not serious	serious [h]	serious [d]	none	**HR 0.80** (0.55 to 1.16)	⊕◯◯◯ VERY LOW
**Breast cancer: IFG/IGT-ADA/WHO**								
4	observational studies	not serious	not serious	not serious	not serious	dose response gradient [j]	**HR 1.19** (1.03 to 1.38)	⊕⊕⊕◯ MODERATE
**Breast cancer: IFG-WHO**								
2	observational studies	not serious	not serious	not serious	not serious	dose response gradient [j]	**HR 1.13** (0.95 to 1.35)	⊕⊕⊕◯ MODERATE

Certainty assessment							Effect	Certainty
No of studies	Study design	Risk of bias	Inconsistency	Indirectness	Imprecision	Other considerations	Summary HR (95% confidence interval)	
**Pancreatic cancer: IFG/IGT-ADA/WHO**								
5	observational studies	not serious	not serious	not serious	not serious	none	**HR 1.22** (1.11 to 1.34)	⨁⨁◯◯ LOW
**Pancreatic cancer: IFG-ADA**								
3	observational studies	not serious	not serious	not serious	not serious	none	**HR 1.25** (1.12 to 1.39)	⨁⨁◯◯ LOW
**Mental/cognitive outcomes**								
**Depressive symptoms: IFG/IGT/HbA1c-ADA/WHO**								
4	observational studies	not serious	not serious	not serious	serious [d]	none	**HR 1.07** (0.80 to 1.43)	⨁◯◯◯ VERY LOW
**Depressive symptoms: IFG-ADA**								
2	observational studies	not serious	not serious	not serious	serious [d]	none	**HR 0.91** (0.67 to 1.23)	⨁◯◯◯ VERY LOW
**All-cause dementia: IFG/IGT-ADA/WHO**								
9	observational studies	not serious	not serious	not serious	not serious	dose response gradient [a]	**HR 1.18** (1.02 to 1.36)	⨁⨁⨁◯ MODERATE
**All-cause dementia: IFG**								
na	observational studies	not serious	not serious	not serious	not serious	dose response gradient [a]	**HR 1.27** (1.08 to 1.49)	⨁⨁⨁◯ MODERATE

Certainty assessment							Effect	Certainty
No of studies	Study design	Risk of bias	Inconsistency	Indirectness	Imprecision	Other considerations	Summary HR (95% confidence interval)	
**All-cause dementia: IGT**								
na	observational studies	not serious	not serious	not serious	not serious	dose response gradient [a]	**HR 1.40** (1.03 to 1.91)	⊕⊕⊕◯ MODERATE
**Alzheimer's dementia: IFG/IGT**								
5	observational studies	not serious	not serious	not serious	not serious	none	**HR 1.36** (1.09 to 1.70)	⊕⊕◯◯ LOW
**Vascular dementia: IFG/IGT**								
3	observational studies	not serious	not serious	not serious	not serious	none	**HR 1.47** (1.01 to 2.15)	⊕⊕◯◯ LOW
**Cognitive impairment: IFG/IGT**								
5	observational studies	not serious	not serious	not serious	not serious	none	**HR 0.96** (0.85 to 1.09)	⊕⊕◯◯ LOW

ADA, American Diabetes Association; CHD, coronary heart disease; CI, confidence interval; CV, cardiovascular; CVD, cardiovascular disease; HR: Hazard ratio, IDF, International Diabetes Federation; IFG, impaired asting glucose; IGT, impaired glucose tolerance; MACE, major adverse cardiac events; TIA, transient ischemic attack; WHO, World Health Organization.

a: upgraded: stronger association for IGT than for IFG
b: upgraded: stronger association for IFG-WHO than for IFG-ADA
c: upgraded: dose-response gradient shown in Cai, 2020 [114]
d: downgraded: 95% CI includes the null value and includes important benefit (SHR ≤0.75) and/or harm benefit (SHR≥1.25)
e: upgraded: dose-response gradient shown in Mitsios, 2018 [117]
f: upgraded: dose-response gradient shown in Aune, 2017 [118]
g: upgraded: stronger association for IFG-WHO than for IFG-WHO and IFG-ADA combined
h: downgraded: mixed outcome were analysed
i: upgraded: strong association: SHR>2
j: upgraded: stronger association for IFG/IGT than for IFG alone

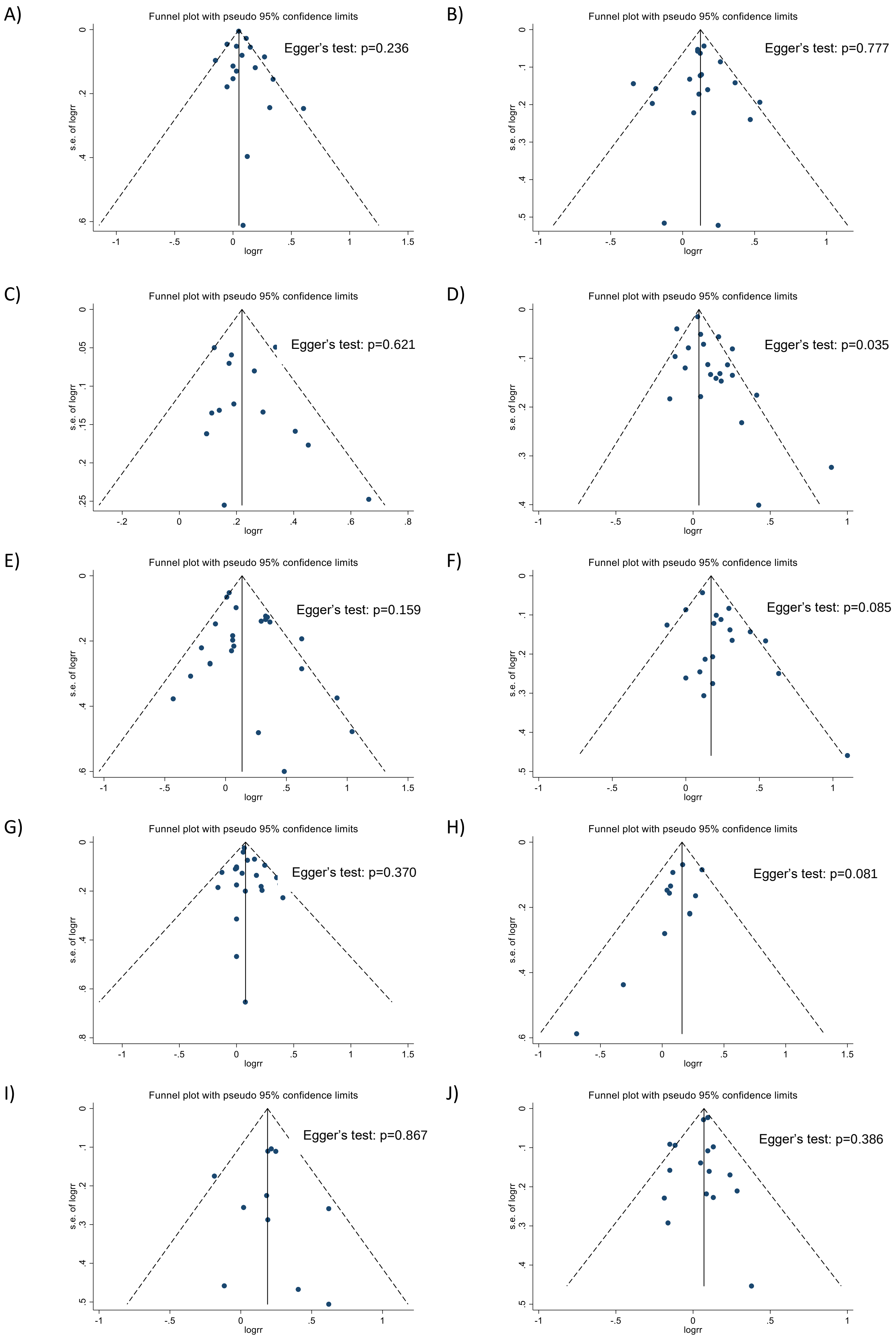
A)
Funnel plot with pseudo 95% confidence limits
Egger's test: p=0.236
B)
Funnel plot with pseudo 95% confidence limits
Egger's test: p=0.777
C)
Funnel plot with pseudo 95% confidence limits
Egger's test: p=0.621
D)
Funnel plot with pseudo 95% confidence limits
Egger's test: p=0.035
E)
Funnel plot with pseudo 95% confidence limits
Egger's test: p=0.159
F)
Funnel plot with pseudo 95% confidence limits
Egger's test: p=0.085
G)
Funnel plot with pseudo 95% confidence limits
Egger's test: p=0.370
H)
Funnel plot with pseudo 95% confidence limits
Egger's test: p=0.081
I)
Funnel plot with pseudo 95% confidence limits
Egger's test: p=0.867
J)
Funnel plot with pseudo 95% confidence limits
Egger's test: p=0.386
s.e. of logrr
logrr

K)

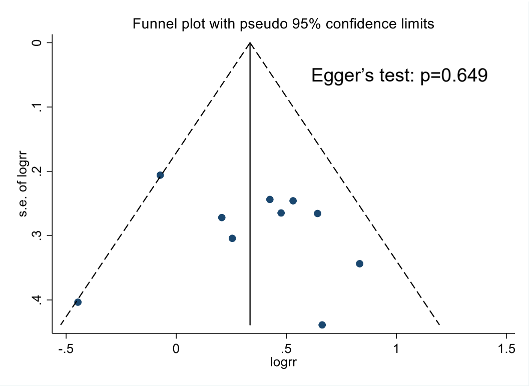

L)

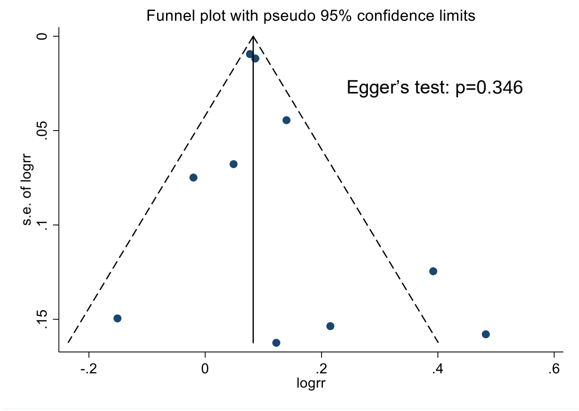

ESM Fig. 1: Funnel plots for the association between prediabetes and A) all-cause mortality (IFG-ADA), B) all-cause mortality (IFG-WHO), C) all-cause mortality (IGT), D) cardiovascular events (IFG-ADA), E) cardiovascular events (IFG-WHO), F) cardiovascular events (IGT), G) coronary heart disease (IFG-ADA), H) coronary heart disease (IFG-WHO), I) coronary heart disease (IGT), J) stroke (IFG-ADA), K) Major adverse cardiac events (MACE) in patients after percutaneous coronary intervention (PCI) (IFG/IGT/HbA1c), and L) heart failure (IFG-ADA)

References

[1] Atan NAD, Koushki M, Motedayen M, et al. (2017) Type 2 diabetes mellitus and non-alcoholic fatty liver disease: a systematic review and meta-analysis. Gastroenterology & Hepatology from Bed to Bench 10: 1-7

[2] Aune D, Schlesinger S, Neuenschwander M, et al. (2018) Diabetes mellitus, blood glucose and the risk of heart failure: A systematic review and meta-analysis of prospective studies. Nutrition, metabolism, and cardiovascular diseases : NMCD 28(11): 1081-1091. 10.1016/j.numecd.2018.07.005

[3] Aune D, Schlesinger S, Norat T, Riboli E (2018) Diabetes mellitus and the risk of abdominal aortic aneurysm: A systematic review and meta-analysis of prospective studies. Journal of diabetes and its complications 32(12): 1169-1174. 10.1016/j.jdiacomp.2018.09.009

[4] Boyle P, Koechlin A, Pizot C, et al. (2013) Blood glucose concentrations and breast cancer risk in women without diabetes: a meta-analysis. European journal of nutrition 52(5): 1533-1540. 10.1007/s00394-012-0460-z

[5] Cavero-Redondo I, Peleteiro B, Alvarez-Bueno C, Rodriguez-Artalejo F, Martinez-Vizcaino V (2017) Glycated haemoglobin A1c as a risk factor of cardiovascular outcomes and all-cause mortality in diabetic and nondiabetic populations: a systematic review and meta-analysis. BMJ open 7(7). 10.1136/bmjopen-2017-015949

[6] Cooper C, Sommerlad A, Lyketsos CG, Livingston G (2015) Modifiable predictors of dementia in mild cognitive impairment: a systematic review and meta-analysis. The American journal of psychiatry 172(4): 323-334. 10.1176/appi.ajp.2014.14070878

[7] de Beer JC, Liebenberg L (2014) Does cancer risk increase with HbA1c, independent of diabetes? British journal of cancer 110(9): 2361-2368. 10.1038/bjc.2014.150

[8] Einarson TR, Machado M, Hemels MEH (2011) Blood glucose and subsequent cardiovascular disease: update of a meta-analysis. Current medical research and opinion 27(11): 2155-2163. 10.1185/03007995.2011.626760

[9] Feinkohl I, Winterer G, Pischon T (2017) Diabetes is associated with risk of postoperative cognitive dysfunction: A meta-analysis. Diabetes/metabolism research and reviews 33(5). 10.1002/dmrr.2884

[10] Geng J, Zhang Y, Wang B, Xie J, Xu B, Li J (2017) Glycosylated hemoglobin levels and clinical outcomes in nondiabetic patients with coronary artery disease: A meta-analysis. Medicine 96(17): e6784. 10.1097/md.0000000000006784

[11] Han H, Zhang T, Jin Z, et al. (2017) Blood glucose concentration and risk of liver cancer: systematic review and meta-analysis of prospective studies. Oncotarget 8(30): 50164-50173. 10.18632/oncotarget.16816

[12] Hsu YT, Nair M, Angelov N, Lalla E, Lee CT (2019) Impact of diabetes on clinical periodontal outcomes following non-surgical periodontal therapy. Journal of clinical periodontology 46(2): 206-217. 10.1111/jcpe.13044

[13] Jayedi A, Djafarian K, Rezagholizadeh F, Mirzababaei A, Hajimohammadi M, Shab-Bidar S (2018) Fasting blood glucose and risk of prostate cancer: A systematic review and meta-analysis of dose-response. Diabetes & metabolism 44(4): 320-327. 10.1016/j.diabet.2017.09.004

[14] Kaminski MR, Raspovic A, McMahon LP, et al. (2015) Risk factors for foot ulceration and lower extremity amputation in adults with end-stage renal disease on dialysis: a systematic review and meta-analysis. Nephrology, dialysis, transplantation : official publication of the European Dialysis and Transplant Association - European Renal Association 30(10): 1747-1766. 10.1093/ndt/gfv114

[15] Kikuta K, Masamune A, Shimosegawa T (2015) Impaired glucose tolerance in acute pancreatitis. World journal of gastroenterology 21(24): 7367-7374. 10.3748/wjg.v21.i24.7367

[16] Koye DN, Shaw JE, Reid CM, Atkins RC, Reutens AT, Magliano DJ (2017) Incidence of chronic kidney disease among people with diabetes: a systematic review of observational studies. Diabetic Medicine 34(7): 887-901. 10.1111/dme.13324

[17] Lau LH, Lew J, Borschmann K, Thijs V, Ekinci EI (2019) The prevalence of Diabetes and its effects on Stroke Outcomes; a meta-analysis and literature review. Journal of diabetes investigation 10(3): 780-792. 10.1111/jdi.12932

[18] Li J, Shao YH, Gong YP, Lu YH, Liu Y, Li CL (2014) Diabetes mellitus and dementia - a systematic review and meta-analysis. European review for medical and pharmacological sciences 18(12): 1778-1789

[19] Liu Y, Yang YM, Zhu J, Tan HQ, Liang Y, Li JD (2011) Prognostic significance of hemoglobin A1c level in patients hospitalized with coronary artery disease. A systematic review and meta-analysis. Cardiovascular diabetology 10: 98. 10.1186/1475-2840-10-98

[20] Miller IM, Skaaby T, Ellervik C, Jemec GB (2013) Quantifying cardiovascular disease risk factors in patients with psoriasis: a meta-analysis. The British journal of dermatology 169(6): 1180-1187. 10.1111/bjd.12490

[21] Mistry BD, Alavi A, Ali S, Mistry N (2017) A systematic review of the relationship between glycemic control and necrobiosis lipoidica diabeticorum in patients with diabetes mellitus. International journal of dermatology 56(12): 1319-1327. 10.1111/ijd.13610

[22] Monje A, Catena A, Borgnakke WS (2017) Association between diabetes mellitus/hyperglycaemia and peri-implant diseases: Systematic review and meta-analysis. Journal of clinical periodontology 44(6): 636-648. 10.1111/jcpe.12724

[23] Pal K, Mukadam N, Petersen I, Cooper C (2018) Mild cognitive impairment and progression to dementia in people with diabetes, prediabetes and metabolic syndrome: a systematic review and meta-analysis. Social psychiatry and psychiatric epidemiology 53(11): 1149-1160. 10.1007/s00127-018-1581-3

[24] Qi W, Zhang N, Korantzopoulos P, et al. (2017) Serum glycated hemoglobin level as a predictor of atrial fibrillation: A systematic review with meta-analysis and meta-regression. PloS one 12(3): e0170955. 10.1371/journal.pone.0170955

[25] Santos-Oliveira R, Purdy C, da Silva MP, dos Anjos Carneiro-Leão AM, Machado M, Einarson TR (2011) Haemoglobin A1c levels and subsequent cardiovascular disease in persons without diabetes: a meta-analysis of prospective cohorts. Diabetologia 54(6): 1327-1334. 10.1007/s00125-011-2078-8

[26] Sarwar N, Aspelund T, Eiriksdottir G, et al. (2010) Markers of dysglycaemia and risk of coronary heart disease in people without diabetes: Reykjavik prospective study and systematic review. PLoS medicine 7(5): e1000278. 10.1371/journal.pmed.1000278

[27] Shi J, Xiong L, Li J, et al. (2015) A Linear Dose-Response Relationship between Fasting Plasma Glucose and Colorectal Cancer Risk: Systematic Review and Meta-analysis. Scientific reports 5: 17591. 10.1038/srep17591

[28] Tan J, You Y, Guo F, Xu J, Dai H, Bie P (2017) Association of elevated risk of pancreatic cancer in diabetic patients: A systematic review and meta-analysis. Oncology letters 13(3): 1247-1255. 10.3892/ol.2017.5586

[29] Williams MF, London DA, Husni EM, Navaneethan S, Kashyap SR (2016) Type 2 diabetes and osteoarthritis: a systematic review and meta-analysis. Journal of diabetes and its complications 30(5): 944-950. 10.1016/j.jdiacomp.2016.02.016

[30] Xu J, Ye Y, Wu H, et al. (2016) Association between markers of glucose metabolism and risk of colorectal cancer. BMJ open 6(6): e011430. 10.1136/bmjopen-2016-011430

[31] Zhong GC, Ye MX, Cheng JH, Zhao Y, Gong JP (2016) HbA1c and Risks of All-Cause and Cause-Specific Death in Subjects without Known Diabetes: A Dose-Response Meta-Analysis of Prospective Cohort Studies. Scientific reports 6: 24071. 10.1038/srep24071

[32] Abudawood M (2019) Diabetes and cancer: A comprehensive review. Journal of Research in Medical Sciences 24. 10.4103/jrms.JRMS_242_19

[33] Akhtar S, Nasir JA, Abbas T, Sarwar A (2019) Diabetes in Pakistan: A systematic review and meta-analysis. Pakistan journal of medical sciences 35(4): 1173-1178. 10.12669/pjms.35.4.194

[34] Jiang X, Zhu Y, Liu Z, Tian Z, Zhu S (2020) Association between diabetes and dental implant complications: a systematic review and meta-analysis. Acta Odontol Scand: 1-10. 10.1080/00016357.2020.1761031

[35] Li X, Li L, Xing Y, Cheng T, Ren S, Ma H (2020) Diabetes Mellitus Is Associated with a Lower Risk of Gout: A Meta-Analysis of Observational Studies. J Diabetes Res 2020: 5470739. 10.1155/2020/5470739

[36] Pan W, Lu H, Lian B, Liao P, Guo L, Zhang M (2019) Prognostic value of HbA1c for in-hospital and short-term mortality in patients with acute coronary syndrome: a systematic review and meta-analysis. Cardiovasc Diabetol 18(1): 169. 10.1186/s12933-019-0970-6

[37] Wang J, Luo X, Jin X, et al. (2020) Effects of Preoperative HbA1c Levels on the Postoperative Outcomes of Coronary Artery Disease Surgical Treatment in Patients with Diabetes Mellitus and Nondiabetic Patients: A Systematic Review and Meta-Analysis. J Diabetes Res 2020: 3547491. 10.1155/2020/3547491

[38] Alatawi Z, Mirghani H (2020) The Association Between Glycemic Variability and Myocardial Infarction: A Review and Meta-Analysis of Prospective Studies and Randomized Trials. Cureus 12(11): e11556. 10.7759/cureus.11556

[39] Bao Y, Gu D (2021) Glycated Hemoglobin as a Marker for Predicting Outcomes of Patients With Stroke (Ischemic and Hemorrhagic): A Systematic Review and Meta-Analysis. Front Neurol 12: 642899. 10.3389/fneur.2021.642899

[40] Arnold LW, Wang Z (2014) The HbA1c and all-cause mortality relationship in patients with type 2 diabetes is J-shaped: a meta-analysis of observational studies. The review of diabetic studies : RDS 11(2): 138-152. 10.1900/rds.2014.11.138

[41] Bejan-Angoulvant T, Cornu C, Archambault P, et al. (2015) Is HbA1c a valid surrogate for macrovascular and microvascular complications in type 2 diabetes? Diabetes & metabolism 41(3): 195-201. 10.1016/j.diabet.2015.04.001

[42] Erqou S, Lee CT, Suffoletto M, et al. (2013) Association between glycated haemoglobin and the risk of congestive heart failure in diabetes mellitus: systematic review and meta-analysis. European journal of heart failure 15(2): 185-193. 10.1093/eurjhf/hfs156

[43] Gorst C, Kwok CS, Aslam S, et al. (2015) Long-term Glycemic Variability and Risk of Adverse Outcomes: A Systematic Review and Meta-analysis. Diabetes care 38(12): 2354-2369. 10.2337/dc15-1188

[44] Harding KA, Pushpanathan ME, Whitworth SR, Nanthakumar S, Bucks RS, Skinner TC (2019) Depression prevalence in Type 2 diabetes is not related to diabetes-depression symptom overlap but is related to symptom dimensions within patient self-report measures: a meta-analysis. Diabet Med 36(12): 1600-1611. 10.1111/dme.14139

[45] Song P, Yu J, Chan KY, Theodoratou E, Rudan I (2018) Prevalence, risk factors and burden of diabetic retinopathy in China: a systematic review and meta-analysis. Journal of global health 8(1): 010803. 10.7189/jogh.08.010803

[46] Wang P, Huang R, Lu S, et al. (2015) HbA1c below 7% as the goal of glucose control fails to maximize the cardiovascular benefits: a meta-analysis. Cardiovascular diabetology 14: 124. 10.1186/s12933-015-0285-1

[47] Zheng J, Cheng J, Zhang Q, Qi C, Wang T, Xiao X (2016) Association Between Glycosylated Hemoglobin Level and Cardiovascular Outcomes in Diabetic Patients After Percutaneous Coronary Intervention. Medicine 95(19): e3696. 10.1097/MD.0000000000003696

[48] Zhou ZY, Liu YK, Chen HL, Yang HL, Liu F (2015) HbA1c and Lower Extremity Amputation Risk in Patients With Diabetes: A Meta-Analysis. The international journal of lower extremity wounds 14(2): 168-177. 10.1177/1534734615593190

[49] Iwanicki C, Logomarsino JV (2019) Impaired glucose tolerance, body mass index and respiratory function in patients with cystic fibrosis: A systematic review. The clinical respiratory journal 13(6): 341-354. 10.1111/crj.13019

[50] Shiferaw WS, Akalu TY, Desta M, et al. (2020) Glycated hemoglobin A1C level and the risk of diabetic retinopathy in Africa: A systematic review and meta-analysis. Diabetes Metab Syndr 14(6): 1941-1949. 10.1016/j.dsx.2020.10.003

[51] Liu X, Xu Y, An M, Zeng Q (2019) The risk factors for diabetic peripheral neuropathy: A meta-analysis. PloS one 14(2): e0212574. 10.1371/journal.pone.0212574

[52] Adeloye D, Ige JO, Aderemi AV, et al. (2017) Estimating the prevalence, hospitalisation and mortality from type 2 diabetes mellitus in Nigeria: a systematic review and meta-analysis. BMJ open 7(5): e015424. 10.1136/bmjopen-2016-015424

[53] Barua R, Templeton AJ, Seruga B, Ocana A, Amir E, Ethier JL (2018) Hyperglycaemia and Survival in Solid Tumours: A Systematic Review and Meta-analysis. Clinical oncology (Royal College of Radiologists (Great Britain)) 30(4): 215-224. 10.1016/j.clon.2018.01.003

[54] Hopper I, Billah B, Skiba M, Krum H (2011) Prevention of diabetes and reduction in major cardiovascular events in studies of subjects with prediabetes: meta-analysis of

randomised controlled clinical trials. European journal of cardiovascular prevention and rehabilitation : official journal of the European Society of Cardiology, Working Groups on Epidemiology & Prevention and Cardiac Rehabilitation and Exercise Physiology 18(6): 813-823. 10.1177/1741826711421687

[55] Hussain MA, Bin-Ayeed SA, Saeed OQ, Verma S, Al-Omran M (2016) Impact of diabetes on carotid artery revascularization. Journal of vascular surgery 63(4): 1099-+. 10.1016/j.jvs.2015.12.041

[56] Leal J, Morrow LM, Khurshid W, Pagano E, Feenstra T (2019) Decision models of prediabetes populations: A systematic review. Diabetes, obesity & metabolism 21(7): 1558-1569. 10.1111/dom.13684

[57] Pillinger T, Beck K, Gobjila C, Donocik JG, Jauhar S, Howes OD (2017) Impaired Glucose Homeostasis in First-Episode Schizophrenia: A Systematic Review and Meta-analysis. JAMA psychiatry 74(3): 261-269. 10.1001/jamapsychiatry.2016.3803

[58] Rashidbeygi E, Safabakhsh M, Delshad Aghdam S, Mohammed SH, Alizadeh S (2019) Metabolic syndrome and its components are related to a higher risk for albuminuria and proteinuria: Evidence from a meta-analysis on 10,603,067 subjects from 57 studies. Diabetes & metabolic syndrome 13(1): 830-843. 10.1016/j.dsx.2018.12.006

[59] Stevens JW, Khunti K, Harvey R, et al. (2015) Preventing the progression to Type 2 diabetes mellitus in adults at high risk: A systematic review and network meta-analysis of lifestyle, pharmacological and surgical interventions. Diabetes research and clinical practice 107(3): 320-331. 10.1016/j.diabres.2015.01.027

[60] Zhang X, Gregg EW, Williamson DF, et al. (2010) A1C level and future risk of diabetes: a systematic review. Diabetes care 33(7): 1665-1673. 10.2337/dc09-1939

[61] Akhtar S, Nasir JA, Sarwar A, et al. (2020) Prevalence of diabetes and pre-diabetes in Bangladesh: a systematic review and meta-analysis. BMJ Open 10(9): e036086. 10.1136/bmjopen-2019-036086

[62] Chivese T, Werfalli MM, Magodoro I, et al. (2019) Prevalence of type 2 diabetes mellitus in women of childbearing age in Africa during 2000-2016: a systematic review and meta-analysis. BMJ open 9(5): e024345. 10.1136/bmjopen-2018-024345

[63] Mirahmadizadeh A, Fathalipour M, Mokhtari AM, Zeighami S, Hassanipour S, Heiran A (2020) The prevalence of undiagnosed type 2 diabetes and prediabetes in Eastern Mediterranean region (EMRO): A systematic review and meta-analysis. Diabetes Res Clin Pract 160: 107931. 10.1016/j.diabres.2019.107931

[64] Zhang B, Zhang B, Zhou Z, Guo Y, Wang D (2021) The value of glycosylated hemoglobin in the diagnosis of diabetic retinopathy: a systematic review and Meta-analysis. BMC Endocr Disord 21(1): 82. 10.1186/s12902-021-00737-2

[65] Au Yeung SL, Luo S, Schooling CM (2018) The Impact of Glycated Hemoglobin (HbA1c) on Cardiovascular Disease Risk: A Mendelian Randomization Study Using UK Biobank. Diabetes care 41(9): 1991-1997. 10.2337/dc18-0289

[66] Dawson LP, Fairley JL, Papandony MC, Hussain SM, Cicuttini FM, Wluka AE (2018) Is abnormal glucose tolerance or diabetes a risk factor for knee, hip, or hand osteoarthritis? A systematic review. Seminars in arthritis and rheumatism 48(2): 176-189. 10.1016/j.semarthrit.2018.02.008

[67] Harati H, Zanetti D, Rao A, et al. (2019) No evidence of a causal association of type 2 diabetes and glucose metabolism with atrial fibrillation. Diabetologia 62(5): 800-804. 10.1007/s00125-019-4836-y

[68] Hope C, Robertshaw A, Cheung KL, Idris I, English E (2016) Relationship between HbA1c and cancer in people with or without diabetes: a systematic review. Diabetic medicine : a journal of the British Diabetic Association 33(8): 1013-1025. 10.1111/dme.13031

[69] Lamport DJ, Lawton CL, Mansfield MW, Dye L (2009) Impairments in glucose tolerance can have a negative impact on cognitive function: a systematic research review. Neuroscience and biobehavioral reviews 33(3): 394-413. 10.1016/j.neubiorev.2008.10.008

[70] Monteiro-Soares M, Boyko EJ, Ribeiro J, Ribeiro I, Dinis-Ribeiro M (2012) Predictive factors for diabetic foot ulceration: a systematic review. Diabetes/metabolism research and reviews 28(7): 574-600. 10.1002/dmrr.2319

[71] Santaguida PL, Balion C, Hunt D, et al. (2005) Diagnosis, prognosis, and treatment of impaired glucose tolerance and impaired fasting glucose. HTA Database

[72] van Durme C, van Echteld I, Falzon L, Aletaha D, van der Heijde D, Landewe RB (2014) Cardiovascular Risk Factors and Comorbidities in Patients with Hyperuricemia and/or Gout: A Systematic Review of the Literature. Journal of Rheumatology 41: 9-14. 10.3899/jrheum.140457

[73] Viera AJ (2011) Predisease: when does it make sense? Epidemiologic reviews 33: 122-134. 10.1093/epirev/mxr002

[74] (2020) Prediabetes and Peripheral Neuropathy. Podiatry Management 39(1): 55-56

[75] Papunen S, Mustakallio-Könönen A, Auvinen J, Timonen M, Keinänen-Kiukaanniemi S, Sebert S (2020) The association between diabetes and cognitive changes during aging. Scand J Prim Health Care 38(3): 281-290. 10.1080/02813432.2020.1802140

[76] Kirthi V, Perumbalath A, Brown E, et al. (2021) Prevalence of peripheral neuropathy in pre-diabetes: a systematic review. BMJ Open Diabetes Res Care 9(1). 10.1136/bmjdrc-2020-002040

[77] Wang DQ, Wang L, Wei MM, et al. (2020) Relationship Between Type 2 Diabetes and White Matter Hyperintensity: A Systematic Review. Front Endocrinol (Lausanne) 11: 595962. 10.3389/fendo.2020.595962

[78] Coutinho M, Gerstein HC, Wang Y, Yusuf S (1999) The relationship between glucose and incident cardiovascular events. A metaregression analysis of published data from 20 studies of 95,783 individuals followed for 12.4 years. Diabetes care 22(2): 233-240. 10.2337/diacare.22.2.233

[79] Ford ES, Zhao G, Li C (2010) Pre-diabetes and the risk for cardiovascular disease: a systematic review of the evidence. Journal of the American College of Cardiology 55(13): 1310-1317. 10.1016/j.jacc.2009.10.060

[80] Huang Y, Cai X, Chen P, et al. (2014) Associations of prediabetes with all-cause and cardiovascular mortality: a meta-analysis. Annals of medicine 46(8): 684-692. 10.3109/07853890.2014.955051

[81] Lee M, Saver JL, Hong KS, Song S, Chang KH, Ovbiagele B (2012) Effect of pre-diabetes on future risk of stroke: meta-analysis. BMJ (Clinical research ed) 344: e3564. 10.1136/bmj.e3564

[82] Levitan EB, Song YQ, Ford ES, Liu SM (2004) Is nondiabetic hyperglycemia a risk factor for cardiovascular disease? A meta-analysis of prospective studies. Archives of internal medicine 164(19): 2147-2155. 10.1001/archinte.164.19.2147

[83] Thomas G, Sehgal AR, Kashyap SR, Srinivas TR, Kirwan JP, Navaneethan SD (2011) Metabolic syndrome and kidney disease: a systematic review and meta-analysis. Clinical journal of the American Society of Nephrology : CJASN 6(10): 2364-2373. 10.2215/cjn.02180311

[84] Xu T, Liu W, Cai X, et al. (2015) Risk of Coronary Heart Disease in Different Criterion of Impaired Fasting Glucose: A Meta-Analysis. Medicine 94(40): e1740. 10.1097/MD.0000000000001740

[85] Gujral UP, Jagannathan R, He S, et al. (2021) Association between varying cut-points of intermediate hyperglycemia and risk of mortality, cardiovascular events and chronic kidney disease: a systematic review and meta-analysis. BMJ Open Diabetes Res Care 9(1). 10.1136/bmjdrc-2020-001776

[86] Chen H, Diu Y, Bhowmik D, Birtcher K, Mortazavi A (2009) Meta-Analysis of the Association between HbA1c Change and Macrovascular Event Risk. Diabetes 58: A190-A190

[87] Erqou S, Lee CTC, Suffoletto M, et al. (2012) Association between Glycated Haemoglobin and the Risk of Congestive Heart Failure in Diabetes Mellitus-Systematic Review and Meta-Analysis. Diabetes 61: A78-A78

[88] Hopper IK, Billah B, Skiba MA, Krum H (2010) Prevention of Diabetes and Reduction in Major Cardiovascular Events in Studies of Subjects with Impaired Glucose Tolerance: Meta-analysis of Randomized Controlled Clinical Trials. Circulation 122(21)

[89] Horikawa C, Kodama S, Tajima R, et al. (2012) Effectiveness of Non-Diagnostic Levels of HbA1c or Fasting Plasma Glucose for Diagnosing and Predicting Diabetes Mellitus: Meta-Analysis. Diabetes 61: A366-A366

[90] Thiagarajan Srinivasan B, Khunti K, Gray LJ, et al. (2011) Progression rates from pre-diabetes to Type 2 diabetes: a systematic review and meta-analysis. Diabetic medicine 28: 2-. 10.1111/j.1464-5491.2011.03232_2.x

[91] Papanas N, Maltezos E (2015) Glycated Hemoglobin as a Risk Factor for Lower Extremity Amputations in Diabetes: "Success Is Counted Sweetest". The international journal of lower extremity wounds 14(2): 106-107. 10.1177/1534734615592313

[92] Robinson CA, Abdel-Motagally M (2011) Comment on: Zhang et al. A1C level and future risk of diabetes: a systematic review. Diabetes Care 2010;33:1665-1673. Diabetes care 34(2): e20; author reply e21. 10.2337/dc10-1901

[93] Desai R, Bhuva R, Sachdeva S, et al. (2020) IMPACT OF PREDIABETES ON LONG-TERM OUTCOMES OF PERCUTANEOUS CORONARY INTERVENTION: A SYSTEMATIC REVIEW AND META-ANALYSIS OF 23,019 PROCEDURES. Journal of the American College of Cardiology 75(11): 1362-1362

[94] Chen P, Ong RTH, Tay WT, et al. (2013) A study assessing the association of glycated hemoglobin a1C (HbA1c) associated variants with HbA1c, chronic kidney disease and diabetic retinopathy in populations of asian ancestry. PloS one 8(11): e79767. 10.1371/journal.pone.0079767

[95] Nagai M, Murakami Y, Tamakoshi A, et al. (2017) Fasting but not casual blood glucose is associated with pancreatic cancer mortality in Japanese: EPOCH-JAPAN. Cancer causes & control : CCC 28(6): 625-633. 10.1007/s10552-017-0884-0

[96] Sarwar N, Gao P, Seshasai SR, et al. (2010) Diabetes mellitus, fasting blood glucose concentration, and risk of vascular disease: a collaborative meta-analysis of 102 prospective studies. Lancet (London, England) 375(9733): 2215-2222. 10.1016/s0140-6736(10)60484-9

[97] Schottker B, Rathmann W, Herder C, et al. (2016) HbA(1c) levels in non-diabetic older adults No J-shaped associations with primary cardiovascular events, cardiovascular and all-cause mortality after adjustment for confounders in a meta-analysis of individual participant data from six cohort studies. BMC medicine 14. 10.1186/s12916-016-0570-1

[98] Cavero-Redondo I, Peleteiro B, Álvarez-Bueno C, Rodríguez-Artalejo F, Martínez-Vizcaíno V (2016) Glycosylated haemoglobin as a predictor of cardiovascular events and mortality: a protocol for a systematic review and meta-analysis. BMJ open 6(7): e012229. 10.1136/bmjopen-2016-012229

[99] Jadhakhan F, Marshall T, Gill P (2015) A systematic review investigating the cumulative incidence of chronic kidney disease in young adults with impaired glucose tolerance. Systematic reviews 4(1): 69. 10.1186/s13643-015-0059-6

[100] Shi R, Diao KY, Shi K, et al. (2020) Effect of prediabetes on the long-term all-cause mortality of patients undergoing percutaneous coronary intervention: A protocol for systematic review and meta analysis. Medicine (Baltimore) 99(33): e21623. 10.1097/md.0000000000021623

[101] Chen S, Zhang Q, Dai G, et al. (2016) Association of depression with pre-diabetes, undiagnosed diabetes, and previously diagnosed diabetes: a meta-analysis. Endocrine 53(1): 35-46. 10.1007/s12020-016-0869-x

[102] Nouwen A, Nefs G, Caramlau I, et al. (2011) Prevalence of depression in individuals with impaired glucose metabolism or undiagnosed diabetes: a systematic review and meta-analysis of the European Depression in Diabetes (EDID) Research Consortium. Diabetes care 34(3): 752-762. 10.2337/dc10-1414

[103] Al-Aali KA (2019) Does Prediabetic State Affects Dental Implant Health? A Systematic Review And Meta-Analysis. Bioscience Biotechnology Research Communications 12(4): 844-854. 10.21786/bbrc/12.4/3

[104] Butler AE, English E, Kilpatrick ES, et al. (2020) Diagnosing type 2 diabetes using Hemoglobin A1c: a systematic review and meta-analysis of the diagnostic cutpoint based on microvascular complications. Acta Diabetol. 10.1007/s00592-020-01606-5

[105] Chinnasamy A, Moodie M (2020) Prevalence of Undiagnosed Diabetes and Prediabetes in the Dental Setting: A Systematic Review and Meta-Analysis. Int J Dent 2020: 2964020. 10.1155/2020/2964020

[106] Eleftheriadou A, Williams S, Nevitt S, et al. (2020) The prevalence of cardiac autonomic neuropathy in prediabetes: a systematic review. Diabetologia. 10.1007/s00125-020-05316-z

[107] Laichuthai N, Abdul-Ghani M, Kosiborod M, Parksook WW, Kerr SJ, DeFronzo RA (2020) Newly Discovered Abnormal Glucose Tolerance in Patients With Acute Myocardial Infarction and Cardiovascular Outcomes: A Meta-analysis. Diabetes Care 43(8): 1958-1966. 10.2337/dc20-0059

[108] Zhou JB, Tang XY, Han YP, Luo FQ, Cardoso MA, Qi L (2020) Prediabetes and structural brain abnormalities: Evidence from observational studies. Diabetes Metab Res Rev 36(4): e3261. 10.1002/dmrr.3261

[109] Richter B, Hemmingsen B, Metzendorf MI, Takwoingi Y (2018) Development of type 2 diabetes mellitus in people with intermediate hyperglycaemia. The Cochrane database of systematic reviews 10: CD012661. 10.1002/14651858.CD012661.pub2

[110] Lee CMY, Colagiuri S, Woodward M, et al. (2019) Comparing different definitions of prediabetes with subsequent risk of diabetes: an individual participant data meta-analysis involving 76 513 individuals and 8208 cases of incident diabetes. BMJ Open Diabetes Res Care 7(1): e000794. 10.1136/bmjdrc-2019-000794

[111] Mitsios J, Ekinci E, Mitsios G, Churilov L, Thijs V (2017) Relationship between glycated haemoglobin (HbA1c) and acute stroke risk: a systematic review and meta-analysis. International Journal of Stroke 12: 9-9

[112] Pan Y, Chen W, Wang Y (2019) Prediabetes and Outcome of Ischemic Stroke or Transient Ischemic Attack: A Systematic Review and Meta-analysis. Journal of stroke and cerebrovascular diseases : the official journal of National Stroke Association 28(3): 683-692. 10.1016/j.jstrokecerebrovasdis.2018.11.008

[113] Xue M, Xu W, Ou YN, et al. (2019) Diabetes mellitus and risks of cognitive impairment and dementia: a systematic review and meta-analysis of 144 prospective studies. Ageing research reviews 55: 100944. 10.1016/j.arr.2019.100944

[114] Cai X, Zhang Y, Li M, et al. (2020) Association between prediabetes and risk of all cause mortality and cardiovascular disease: updated meta-analysis. Bmj 370: m2297. 10.1136/bmj.m2297

[115] Li Y, Li XW, Zhang YH, et al. (2020) Prognostic significance of the hemoglobin A1c level in non-diabetic patients undergoing percutaneous coronary intervention: a meta-analysis. Chin Med J (Engl) 133(18): 2229-2235. 10.1097/cm9.0000000000001029

[116] Huang Y, Cai X, Mai W, Li M, Hu Y (2016) Association between prediabetes and risk of cardiovascular disease and all cause mortality: systematic review and meta-analysis. BMJ (Clinical research ed) 355: i5953. 10.1136/bmj.i5953

[117] Mitsios JP, Ekinci EI, Mitsios GP, Churilov L, Thijs V (2018) Relationship Between Glycated Hemoglobin and Stroke Risk: A Systematic Review and Meta-Analysis. Journal of the American Heart Association 7(11): 1-10. 10.1161/JAHA.117.007858

[118] Aune D, Feng T, Schlesinger S, Janszky I, Norat T, Riboli E (2018) Diabetes mellitus, blood glucose and the risk of atrial fibrillation: A systematic review and meta-analysis of cohort studies. Journal of diabetes and its complications 32(5): 501-511. 10.1016/j.jdiacomp.2018.02.004

[119] Aune D, Schlesinger S, Norat T, Riboli E (2018) Diabetes mellitus and the risk of sudden cardiac death: A systematic review and meta-analysis of prospective studies. Nutrition, metabolism, and cardiovascular diseases : NMCD 28(6): 543-556. 10.1016/j.numecd.2018.02.011

[120] Zhao Y, Guo M, Shi G (2020) Prediabetes predicts adverse cardiovascular outcomes after percutaneous coronary intervention: a meta-analysis. Biosci Rep 40(1). 10.1042/bsr20193130

[121] Echouffo-Tcheugui JB, Venkat Narayan KM, Weisman D, Golden SH, Jaar BG (2016) Association between prediabetes and risk of chronic kidney disease: a systematic review and meta-analysis. Diabetic medicine : a journal of the British Diabetic Association 33(12): 1615-1624. 10.1111/dme.13113

[122] Mutie PM, Pomares-Millan H, Atabaki-Pasdar N, et al. (2020) An investigation of causal relationships between prediabetes and vascular complications. Nat Commun 11(1): 4592. 10.1038/s41467-020-18386-9

[123] Huang Y, Cai X, Qiu M, et al. (2014) Prediabetes and the risk of cancer: a meta-analysis. Diabetologia 57(11): 2261-2269. 10.1007/s00125-014-3361-2

[124] Xu WG, Qian YF, Wu J (2017) The effect of prediabetes on hepatocellular carcinoma risk: a systematic review and meta-analysis. Minerva medica 108(2): 185-190. 10.23736/s0026-4806.16.04601-2

[125] Fu DX, Cui HB, Guo NN, Su N, Xu JX, Wang GY (2016) Prediabetes and the risk of pancreatic cancer: A meta-analysis. International journal of clinical and experimental medicine 9(10): 19474-19479

[126] Tong A, Wang X, Li F, Xu F, Li Q, Zhang F (2016) Risk of depressive symptoms associated with impaired glucose metabolism, newly diagnosed diabetes, and previously diagnosed diabetes: a meta-analysis of prospective cohort studies. Acta diabetologica 53(4): 589-598. 10.1007/s00592-016-0845-1

[127] Cai X, Liu X, Sun L, et al. (2021) Prediabetes and the risk of heart failure: A meta-analysis. Diabetes Obes Metab. 10.1111/dom.14388
